# Cooperative expression of atomic chirality in inorganic nanostructures

**DOI:** 10.1038/ncomms14312

**Published:** 2017-02-02

**Authors:** Peng-peng Wang, Shang-Jie Yu, Alexander O Govorov, Min Ouyang

**Affiliations:** 1 Department of Physics and Center for Nanophysics and Advanced Materials, University of Maryland, College Park, Maryland 20742, USA; 2 Department of Electrical and Computer Engineering, University of Maryland, College Park, Maryland 20742, USA; 3 Department of Physics and Astronomy, Ohio University, Athens, Ohio 45701, USA; 4 Institute of Fundamental and Frontier Sciences, University of Electronic Science and Technology of China, Chengdu 610054, China

## Abstract

Cooperative chirality phenomena extensively exist in biomolecular and organic systems via intra- and inter-molecular interactions, but study of inorganic materials has been lacking. Here we report, experimentally and theoretically, cooperative chirality in colloidal cinnabar mercury sulfide nanocrystals that originates from chirality interplay between the crystallographic lattice and geometric morphology at different length scales. A two-step synthetic scheme is developed to allow control of critical parameters of these two types of handedness, resulting in different chiral interplays expressed as observables through materials engineering. Furthermore, we adopt an electromagnetic model with the finite element method to elucidate cooperative chirality in inorganic systems, showing excellent agreement with experimental results. Our study enables an emerging class of nanostructures with tailored cooperative chirality that is vital for fundamental understanding of nanoscale chirality as well as technology applications based on new chiroptical building blocks.

## Abstract (web summary)

Cooperative chirality is common in organic and biomolecular systems but is rarely explored in inorganic materials. Here, the authors combine synthesis and theory to uncover cooperative chirality in inorganic nanocrystals, arising from the interplay of independently-controlled crystallographic and morphological chirality.

Chirality, or handedness, is an important geometric attribute of nature that is observed in its various forms with lack of mirror symmetry^1^,^2^. The significance of chirality of matter has been addressed in various fields, ranging from understanding the evolution of life processes, to enantioselectivity in chemical reactions, to the recent discovery of unusual large spin polarization through chiral organic molecules for spin chemistry and devices^3^,^4^,^5^. The most common examples of chiral entities studied so far are small organic molecules that possess, for example, an asymmetrically substituted carbon atom in an *sp*^3^-hybridized carbon skeleton. When these microscopic monomeric organic chiral units are assembled as building blocks to form higher-order meso-, or macro-scopic aggregates, chirality can be expressed as observables at various length scales via a cooperative effect due to intra- and inter-molecular chirality interactions among primary chiral units^6^,^7^. While such cooperative chirality phenomena extensively exist in the biomolecular world, related studies in inorganic systems have been lacking. As compared with biological and organic systems, inorganic materials can often offer much better control of periodicity and morphology^8^, thus representing unique test beds to understand and tailor cooperative chirality at different length scales. A series of inorganic crystals have been found to possess helical atomic arrangement along certain crystallographic directions, leading to crystallographic chirality in a periodic lattice^9^,^10^,^11^,^12^,^13^. Therefore achieving independent control of critical chiral parameters of both lattice and morphology in an inorganic structure should be the key to understanding and controlling its cooperative chirality, but has posed significant challenges because it requires synthetic control at different length scales. For example, recent attempts to control both lattice and morphology chirality in one-pot synthesis of colloidal selenium and tellurium nanostructures have only led to observation of optical activity dominated by chiral shape with an absence of chiral lattice contribution in both theoretical modelling and experiments^14^.

Herein, we develop a novel epitaxy based two-step synthetic scheme to achieve independent control of crystallographic and geometric chirality in an inorganic nanostructure, and we employ cinnabar α-HgS as an example to demonstrate versatile control and to explore enabled chirality interplay originating from primary atomic lattice and higher-order morphology with in-depth structural and optical characterization. This synthetic paradigm can allow precise tailoring of chirality at different length scales with a high degree of freedom of control by versatile combination of crystallographic and geometric handedness, thus opening up exciting opportunities to study and gain insight into unique cooperative chirality in an inorganic system. By performing systematic circular dichroism (CD) measurements on samples with different combinations of crystallographic and geometric chirality, evolution of cooperative chirality can be revealed at multiple levels of inorganic nanostructures. We further adopt an electromagnetic core-shell model with the finite element method (FEM) that can allow computation and prediction of cooperative chirality, showing excellent agreement with our experimental results and clear elucidation of nanoscale chirality interplay. Importantly, both the synthetic scheme and theoretical model in our current work are universal for exploring cooperative chirality, and can be readily applied for other inorganic materials that possess a chiral symmetry group. This work can therefore enable an emerging class of inorganic nanostructures with pre-designed cooperative chirality that not only allows fundamental understanding and control of chirality at different length scales, but also provides functional building blocks with engineered handedness to achieve chirality-dependent chemical and physical processes as well as large-scale meta-devices with new chiroptical effects^15^,^16^,^17^,^18^.

## Results

### Cooperative chirality in an inorganic nanosystem

**Figure 1:**
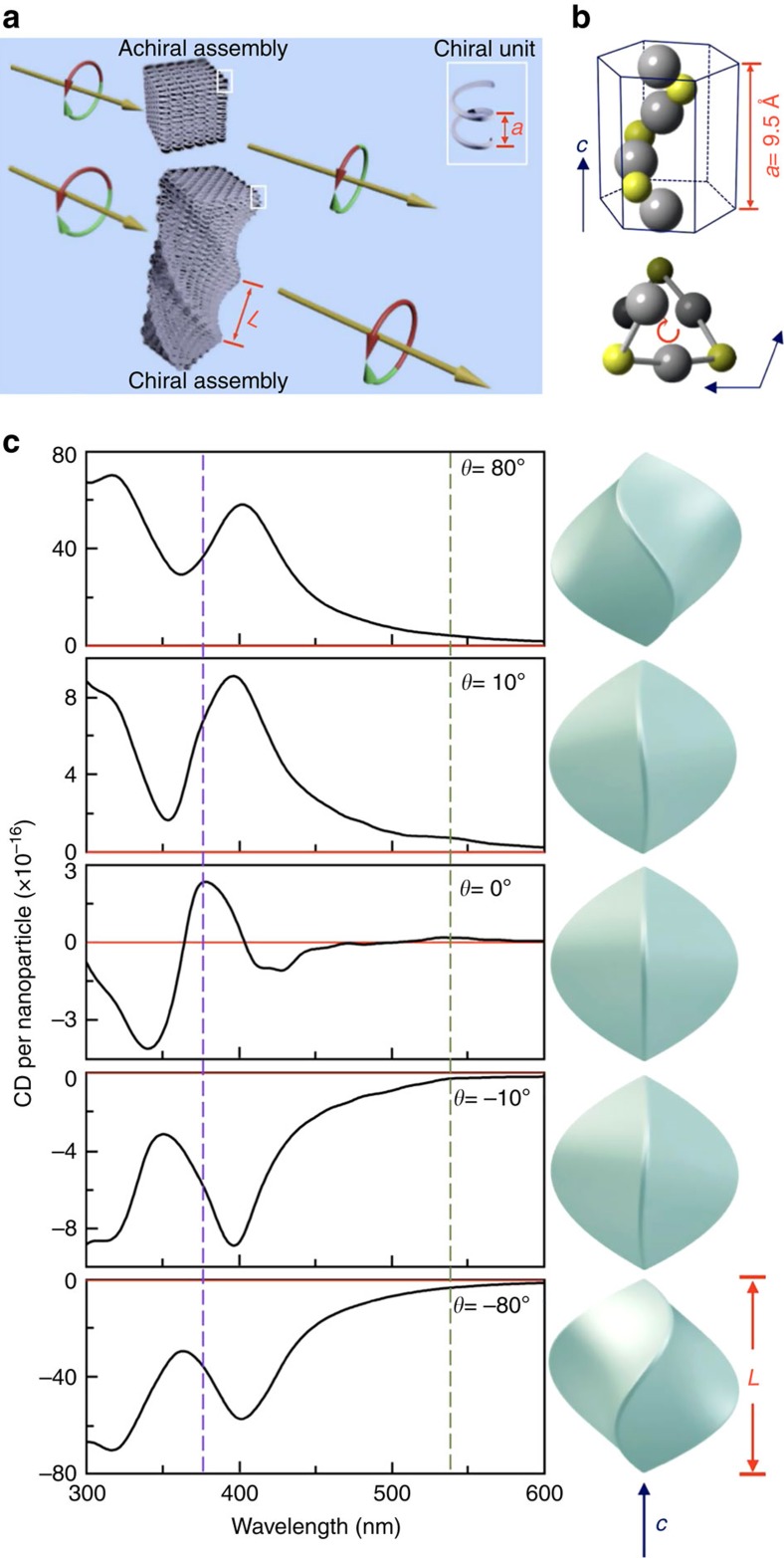
Cooperative chirality at different length scales. (**a**) Schematic model of nanoscale assembly of chiral units with characteristic length *a* (highlighted by white rectangle). Such assemblies can possess either achiral or chiral morphology with characteristic length *L*, leading to different chiroptical response. (**b**) Atomic model of cinnabar HgS lattice along crystallographic *c* axis, showing helical arrangement of atoms. The top image shows the side view, and the bottom image shows the top view. Grey sphere, Hg atom. Yellow sphere, S atom. (**c**) Computed CD spectra of a twisted triangular bipyramid α-HgS with different twisting angle, while the crystallographic chirality remains unchanged. Corresponding structural model of simulated nanostructures is presented next to its CD spectroscopy. The simulation is performed with an incident wave vector that is 30° relative to the *c* axis of nanoparticle. Green and purple dashed lines are guides to the eyes for features near 540 nm and 380 nm, respectively. For all simulations, the *L* and aspect ratio of α-HgS nanostructures are set to be 150 nm and 1, respectively.

A schematic of chirality expressed at different length scales is shown in Fig. 1a, and can be omnipresent in diverse systems (biological, biomimetic and inorganic systems). The primary chiral units can be chiral molecules or chiral unit cells in solid state, and the overall chirality beyond atomic and molecular scale should be considered as various cooperative chiral interactions among primary chiral units. Figure 1b shows one example of cinnabar α-HgS lattice with a space group of *P*3_2_21, in which Hg and S atoms are arranged in a helical form along crystallographic *c* axis^19^. This offers a natural atomic scale primary chiral unit, and the assembly of such atomic scale chiral entity (that is, periodic lattice in solid) is analogous to higher-order biological system and should manifest cooperative chirality. To explore cooperative chirality originating from such crystallographic and geometric effects, we have adopted an electromagnetic model to incorporate both crystallographic and geometric chirality in FEM simulation. Detailed description of our model and FEM simulation is provided in Supplementary Figs 1,2, Supplementary Table 1 and Supplementary Note 1. Briefly, our model and computation of chiroptical response is based on Maxwell's equations with constitutive relations for continuous chiral media. The key parameters describing the property of a chiral media include the dielectric function (*ɛ*) and the chiral parameter (*ξ*), which can be determined via a self-consistent parametric modelling by fitting both experimental CD and extinction measurements acquired from small sized spherical nanoparticles with corresponding analytical solutions (Supplementary Fig. 2). While chiroptical response induced by either lattice or morphology in an inorganic nanostructure has been separately described in existing theoretical work^14^,^20^,^21^, our current computational model can allow evaluation of cooperative CD features originating from both crystallographic and geometric effects at the different length scales. Figure 1c illustrates cooperative CD spectra in the ultraviolet–visible regime by using a twisted triangular bipyramid α-HgS nanostructure as an example to highlight unique opportunity to tailor CD features of inorganic materials at the nanoscale. Our definition of geometric left-(M) and right-(P) handedness of twisted nanostructures follows the convention of helix protein and P/M nomenclature (see also Supplementary Fig. 3 for definition of twisting angle *θ*)^22^,^23^. We have computed and compared CD spectra of a series of M and P nanostructures of α-HgS with different *θ* (see Supplementary Fig. 4 for top view of twisted triangular bipyramid nanostructures), while their size, aspect ratio and crystallographic chirality are kept the same. When the *θ*=0 (this corresponds to achiral morphology with no chiral contribution from geometric morphology), our computation shows that the untwisted triangular bipyramid α-HgS nanostructure manifests two major CD resonances at 540 and 380 nm, which are consistent with previous observation of α-HgS nanoparticles, and can be attributed to the first and higher excitonic transitions that are determined by the periodic chiral lattice, respectively (see Fig. 1b)^21^. This also provides a sanity check of our modelling and simulation. By twisting in morphology along *c* axis, geometric handedness of nanostructure can be introduced at different length scales merely determined by its size. The evolution of cooperative CD features with *θ* clearly shows the interplay between the crystallographic and geometric chirality: First, we have evaluated that the CD response merely contributed from the geometric morphology (this can be achieved by setting the *ξ* to be zero to eliminate the contribution from the chiral lattice), and presented the results in Supplementary Fig. 5. The well-defined CD features can be present even with *ξ*=0. Second, as shown in Supplementary Fig. 5, a larger twisting angle can lead to significantly more pronounced chiral contribution from the morphology. When the *θ* is increased from 10° to 80° (or decreased from −10° to −80° for the right-handed twisting), the morphology induced CD signal can be enlarged by about eight fold. And last, when the twisting orientation is reversed, its geometric CD contribution is opposite and the CD components induced merely by chiral morphology show a mirror relationship (Supplementary Fig. 5). This is in contrast to the resultant overall CD spectra presented in Fig. 1c, in which the overall CD spectra computed from two opposite *θ* are not mirrored spectra after taking into account of both crystallographic and geometric effects. This confirms the cooperative chirality from both chiral lattice and chiral morphology.

### Synthetic control of crystallographic and geometric chirality

**Figure 2:**
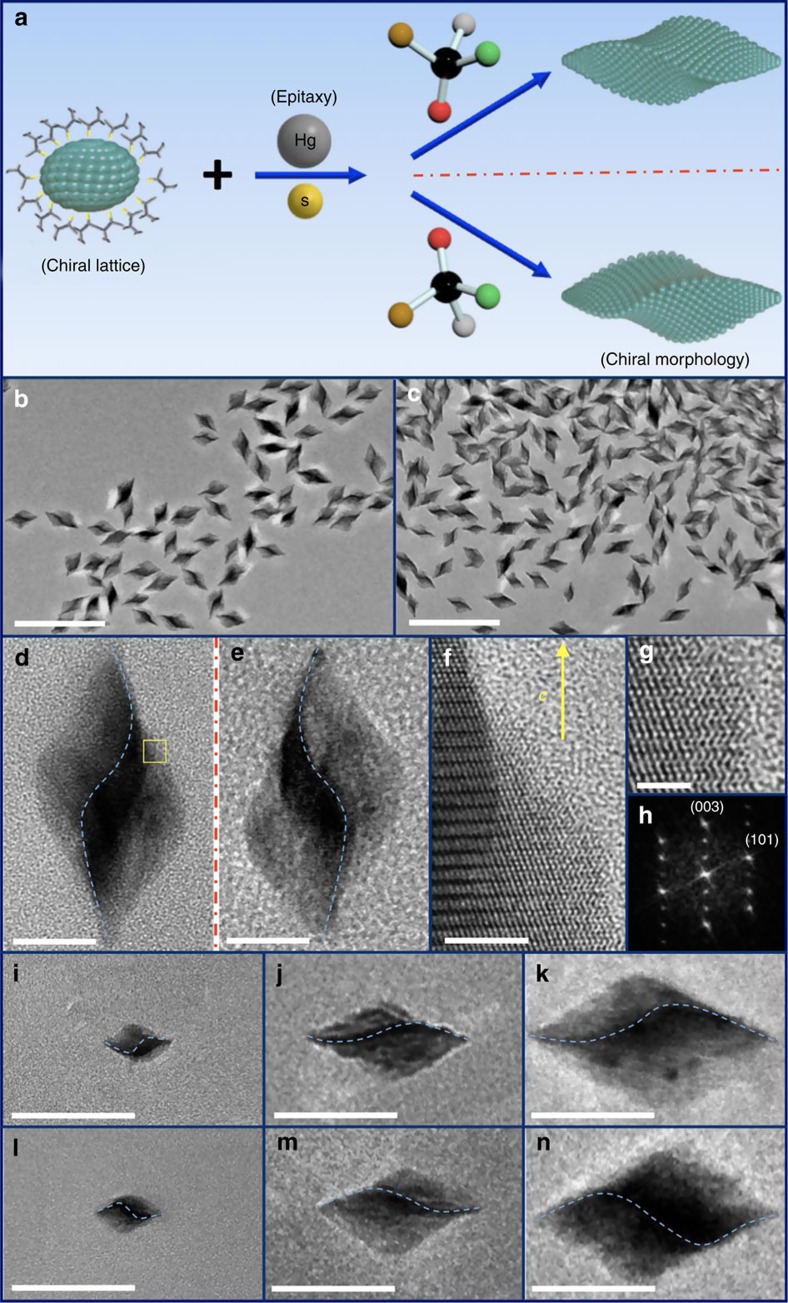
Two-step synthesis for controlling crystallographic and geometric handedness. (**a**) Schematic of the growth process based on epitaxial principle with involvement of chiral molecule to tailor the chirality of morphology. (**b**,**c**) Large-scale TEM image of as-synthesized α-HgS nanostructures with (+)_*C*_-P and (+)_*C*_-M nanostructures by following synthetic route in **a**, respectively. Scale bar, 200 nm. (**d**,**e**) Typical TEM images of prevailing individual nanostructures from **b** and **c**, respectively. Blue dashed curves are added to guide the eyes for different twisting orientation in a nanostructure. Scale bar, 20 nm. (**f**) High-resolution TEM image of selected yellow area in **d**. Helical atomic arrangement along crystallographic *c* axis can be unambiguously identified, as illustrated in Fig. 1b. Scale bar, 5 nm. (**g**) Better resolution to reveal feature of helical atomic arrangement. Scale bar, 2 nm. (**h**) Fourier transform of TEM image, confirming assignment of crystallographic axis. (**i**–**n**) TEM images of (+)_*C*_-M (**i**–**k**) and (+)_*C*_-P (**l**–**n**) α-HgS nanostructures with different size. Blue dashed curve is added to guide the eyes for different twisting orientation in a nanostructure. Scale bar, 100 nm.

Experimentally, while there exist a number of studies on control of either crystallographic or geometric chirality^14^,^21^,^24^,^25^,^26^,^27^,^28^,^29^, simultaneous and independent control of both chiral factors in a nanostructure has posed intimidating synthetic challenges, but represents a prerequisite in order to understand and even control their interplay. We have developed a solution phase two-step synthetic method by combining homo-epitaxial growth with atomic scale regulation of morphology through a chiral molecular modifier, with the key steps of our synthetic control scheme illustrated in Fig. 2a. Briefly, small single crystalline α-HgS nanocrystals with well-defined crystallographic handedness are applied as seeds in the synthesis, followed by slow co-addition of Hg and S precursors to enable successive ion layer adsorption and reaction onto the seeds at the presence of excess enantiopure molecules as chiral molecular modifiers of morphology^30^. Because of the epitaxial synthetic condition, the seed nanoparticle acts as a chiral lattice template, in which the addition of precursor atoms follows the existing chiral lattice structure defined by the seeds, therefore determining the crystallographic chirality of epitaxially grown nanostructures. However, the addition of surrounding chiral molecules (such as *D*- or *L*-penicillamine molecules) in solution can play a pivotal role in surface reconstruction/reshaping, by acting as molecular chiral modifier through the interaction with surface atoms during the epitaxial growth to transmit their handedness into the morphology of nanostructures and to allow independent geometric control at larger length scale. Surface reconstruction enabled by different chiral molecules has been observed in various two-dimensional surfaces^31^,^32^,^33^,^34^,^35^,^36^,^37^,^38^,^39^,^40^, however, the application of chiral molecules in an epitaxial solution growth process to directly enable geometric morphology control of colloidal nanostructures is new.

Following the synthetic scheme illustrated in Fig. 2a, we have started with small α-HgS nanocrystals as seeds by a modified enantioselective synthesis^21^. The α-HgS seeds from this enantioselective synthesis can possess different lattice chirality with the same achiral morphology, which show a mirrored CD response (see Supplementary Fig. 6), and are denoted as (+)_*C*_ and (−)_*C*_ based on their CD features for the rest of discussion to represent the two different crystallographic handedness, respectively. In the succeeding epitaxial growth process, we have utilized *D*- and *L*-penicillamine molecules as exemplary molecular chiral modifiers to demonstrate control of morphological chirality, but different chiral molecules can also be available and employed to maximize such synthetic control. Figure 2b,c show two typical large-scale transmission electron microscopy (TEM) images of epitaxially grown α-HgS nanostructures, which are grown from the same seed nanoparticles possessing crystallographic lattice chirality of (+)_*C*_, but with the incorporation of two penicillamine enantiomers as chiral morphology modifiers during the epitaxial growth process, respectively. Both results show that the addition of penicillamine molecules in the epitaxial growth process has unanimously led to twisted triangular bipyramid nanostructures with narrow size distribution (average length and aspect ratio of nanostructures in both samples are 76.9±5.8 nm and 1.90±0.15, respectively). However, the prevailing handedness of twisting in morphology from both samples is different. Two high-resolution TEM images are presented and compared in Fig. 2d,e with highlight dominant morphology from the samples in Fig. 2b,c, respectively. A few features can be immediately identified: first, both samples show twisted triangular bipyramid morphology. To further confirm such triangular bipyramid structures we have performed three-dimensional (3D) tomographic imaging and reconstruction in TEM with side and top views shown in Supplementary Movies 1 and 2, respectively. A clear three-fold symmetry and twisted structure can be identified. By comparing with structural modelling, the twisting angle can be determined to be *θ*= 65.3±3.5° (Supplementary Figs 3 and 7). Second, even though these two samples were grown from the same chiral (+)_*C*_ -seed nanoparticles, their twisting orientations (that is, geometric handedness) are prevailingly different, and their morphologies show non-superimposable 3D mirror relationship (Fig. 2d,e). On the basis of the twisting direction relative to the *c* axis, we can assign the geometric handedness of structures in Fig. 2d,e as P and M, respectively. Third, our X-ray diffraction characterization (Supplementary Fig. 8) confirms the as-synthesized nanostructures possess cinnabar lattice structures. Figure 2f,g show atomic resolution images and its corresponding Fourier Transform image (Fig. 2h), determining the long axis of twisted nanoparticles is along *c* axis of cinnabar structure and, importantly, the helical lattice arrangement as schematically shown in Fig. 1b is unambiguously revealed by HRTEM for the cinnabar lattice. And last, we have also performed similar epitaxial synthesis with (−)_*C*_-seed nanoparticles possessing opposite crystallographic chirality (that is, (−)_*C*_-M and (−)_*C*_-P), and presented the results in Supplementary Fig. 9. We have found that the prevailing morphology from similar epitaxial synthesis is indeed determined by the chiral molecules utilized during the epitaxial growth and is independent of the crystallographic chirality of the seed nanoparticles. Our synthetic scheme in Fig. 2a can therefore allow independent control of crystallographic and geometric handedness, by controlling the seeds and the follow-up epitaxial growth in a two-step solution process.

Other than morphology and its associated geometric handedness, our synthetic scheme can also allow important size control by simply manoeuvreing the growth condition (see ‘Methods' section and Supplementary Table 2). For example, by controlling the amount of seed nanoparticles and precursors in an epitaxial synthesis, the size of nanoparticles with well-defined handedness can be continuously tuned from 30 to 270 nm along the *c* axis. Figure 2i–n exemplify such size evolution for both M (Fig. 2i–k) and P (Fig. 2l–n) morphology, with corresponding large-scale images shown in Supplementary Fig. 10 to highlight the uniformity of as-synthesized structures. One important structural feature that we have observed from this size evolution is that while the overall size of the twisted nanoparticles varies, both of their aspect ratio and *θ* remains essentially unchanged (see Supplementary Table 3). To that, the synthetic scheme in Fig. 2a can offer utmost control of structural parameters critical to explore and tailor the cooperative chirality of nanostructures through the handedness combination of crystallographic lattice and geometric morphology.

**Figure 3:**
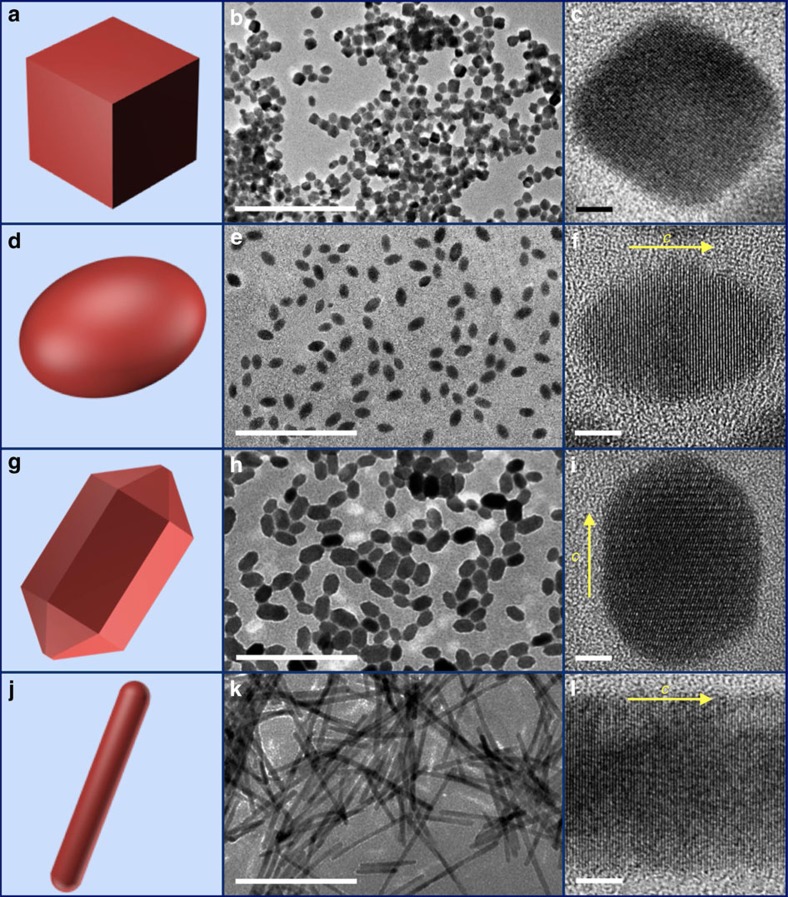
The α-HgS nanostructures with chiral lattice ((+)_*C*_) but various achiral morphology. (**a**–**c**) Model, typical large-scale TEM image and high-resolution TEM image of nanocubes, respectively. (**d**–**f**) Model, typical large-scale TEM image and high-resolution TEM image of nanoellipsoids, respectively. (**g**–**i**) Model, typical large-scale TEM image and high-resolution TEM image of nanorods, respectively. (**j**–**l**) Model, typical large-scale TEM image and high-resolution TEM image of nanowires (denoted as ‘Nanowires 1'), respectively. More nanowires with different aspect ratio are provided in Supplementary Fig. 12. The yellow arrow in **f**,**i** and **l** represents crystallographic *c* axis of α-HgS. Scale bar for large-scale TEM images, 200 nm. Scale bar for high-resolution TEM images is 5 nm.

To illustrate the effect of geometric morphology on cooperative chirality, we have also synthesized a series of α-HgS nanostructures possessing crystallographic chirality but different achiral morphology, with four examples (nanocubes, nanoellipsoids, nanorods and nanowires) highlighted in Fig. 3. All the nanostructures summarized in Fig. 3 possess uniform size and well-defined achiral morphology without twisting, which are different from those in Fig. 2. High-resolution TEM images and powder X-ray diffraction spectra (Supplementary Fig. 11) of all nanostructures in Fig. 3 have confirmed cinnabar lattice. For nanoellipsoids, nanorods and nanowires, the inter-planar lattice spacing along the long axis corresponds to that of (003) plane of α-HgS, supporting our assignment of their long axis as the *c* axis of cinnabar. Similar to nanostructures with chiral morphology in Fig. 2, the size of HgS nanostructures with achiral morphology can also be tuned with a few more examples shown in Supplementary Fig. 12. Together with the chiral morphologies presented in Fig. 2, all our synthetic controls achieved so far can allow systematic study of the evolution of cooperative CD with critical structural parameters and can facilitate our understanding of the interplay between crystallographic and geometric handedness in the chiroptical properties of inorganic nanostructures.

### Interplay between crystallographic and geometric chirality

**Figure 4:**
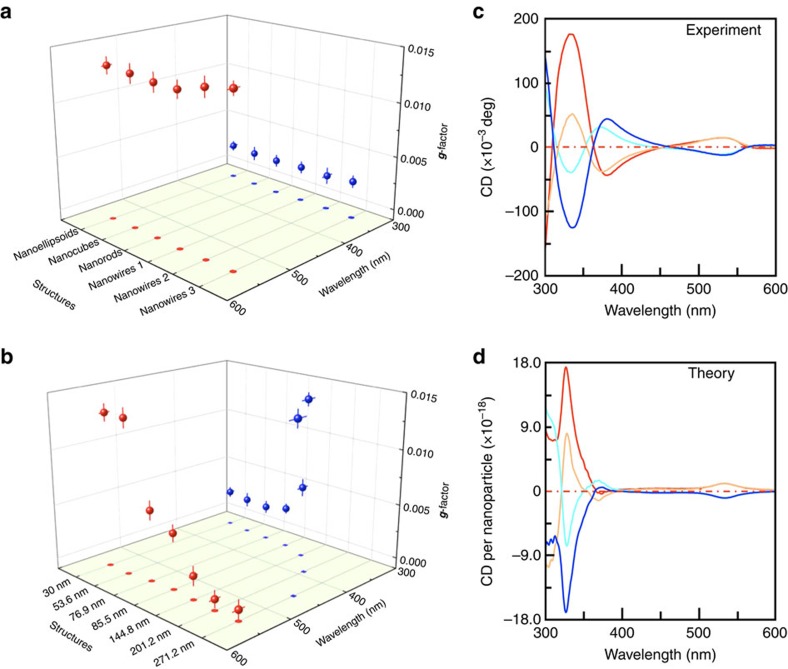
Chiroptical response of α-HgS nanostructures with interplay between crystallographic and geometric handedness. (**a**) Summary of ***g*** factors of α-HgS nanoparticles, possessing chiral (+)_*C*_-lattice but different achiral morphology. (**b**) Wavelength dependent evolution of ***g***-factor peaks with size for the (+)_*C*_-M twisted triangular bipyramid α-HgS nanoparticles. In (**a**,**b**) projections of the ***g*** factors onto the basal plane are also presented to highlight wavelength shifting of peak position. (**c**) Comparison of CD spectra of 85 nm long twisted bipyramid α-HgS nanoparticles with different combination of crystallographic and geometric handedness. Red, (+)_*C*_-M. Orange, (+)_*C*_-P. Cyan, (−)_*C*_-M. Blue, (−)_*C*_-P. (**d**) Computed CD spectra to compare with **c**. Colour codes are assigned to be the same as those in **c**. The experimental dissymmetric ***g***-factor and its associated error bar are determined as average and standard deviation of statistical analysis over different sample batches and different runs of measurement, respectively.

We have performed and compared ultravilolet–visible CD and extinction spectra measurements on samples with different combinations of crystallographic lattice and geometric morphology features that are exemplified in Figs 2 and 3. We have first investigated the CD spectra of α-HgS nanostructures presented in Fig. 3 and Supplementary Fig. 12, in which all samples possess the same chiral lattice, (+)_*C*_, but different achiral morphologies and aspect ratios, and presented the results in Supplementary Figs 13 and 14. Qualitatively, we have found that all α-HgS nanoparticles with different achiral morphologies possess similar CD features in the ultraviolet–visible wavelength regime. For all nanostructures with achiral morphology, two distinct CD peaks occur at the same wavelengths as those from seed nanoparticles, respectively, and can be assigned to be associated with different excitonic states in the chiral lattice^21^. For the α-HgS nanostructures with the same achiral morphology, their CD spectra can be reversed by changing the handedness of their lattice (Supplementary Fig. 15). To gain more quantitative understanding of their chiroptical properties, we have evaluated and compared the dissymmetric factor ***g***, among different achiral morphologies with same crystallographic handedness. The dissymmetric factor ***g*** is defined as the CD spectra normalized by its corresponding extinction^5^. This ***g*** factor is a dimensionless quantity that represents the chiroptical response per one HgS molecular unit, and can be utilized to compare among different chiral nanoparticles with elimination of a few disorder effects, including variations of nanoparticles' concentration and size^20^,^21^. Figure 4a summarizes the dissymmetric ***g*** factors at different wavelengths for α-HgS nanostructures with different achiral morphologies and aspect ratios. It can be clearly seen that when the achiral morphology or the structural aspect ratio varies, both magnitude and central wavelength of two CD peaks remain essentially unchanged. This observation further confirms that the observed CD features of α-HgS nanostructures with achiral morphology indeed originate from crystallography rather than other effects, which is also consistent with our definition and understanding of ***g*** factors of nanoparticle that represent chiroptical response per one HgS molecular unit. We have further computed CD and extinction spectra of (+)_*C*_-HgS nanostructures with all different achiral morphology summarized in Fig. 3 and Supplementary Fig. 12, and compared them with experimental results (see Supplementary Figs 13 and 14). Agreement between the experimental and theoretical results confirms negligible contribution from an achiral geometry on overall chirality, and substantiates our understanding of characteristics of ***g*** factors in chiral nanostructures with intrinsic crystallographic handedness.

We have also performed similar CD measurements on as-synthesized twisted triangular bipyramid α-HgS nanostructures in Fig. 2 that possess both crystallographic and geometric handedness (Supplementary Fig. 16), and summarized the dependence of their corresponding dissymmetric ***g*** factors on both wavelength and size of nanostructures (the length *L* of nanostructures along their *c* axis is used to represent their size in the figure) in Fig. 4b. By comparing with Fig. 4a, a few prominent features can be immediately identified in Fig. 4b: when the size of chiral nanostructures increases, the peak position of the first ***g***-factor (red dots) remains almost unchanged, centring at ∼540 nm. However, the peak position of the second ***g***-factor (blue dots) is red-shifted in the spectra. In the meantime, the peak magnitude of the second ***g***-factor increases as the size increases, while the peak magnitude of the first ***g***-factor peaks manifests the opposite tendency. Distinct variation of the tendency of these two ***g*** factors suggests that their physical origins are different. Similar to nanostructures with different achiral morphologies, the ***g***-factor at 540 nm can be assigned to crystallographic chirality and is independent on morphology. On the other hand, chiral morphology can induce an additional CD response in the ultraviolet–visible regime, which is qualitatively related to the characteristic length *L* of the twisted shape (Fig. 1a). Therefore the corresponding CD response of such twisted nanostructures in the ultraviolet–visible regime represents competition between crystallographic and geometric handedness. As a result, when the size of nanostructures increases (thus, the *L* increases), the ***g***-factor peak in this wavelength regime red-shifts (an additional electromagnetic phase retardation effect can further contribute to the red-shifting of the second ***g***-factor in the spectra^41^). In addition to the variation of central peak position, an increase of the size of nanostructures with chiral morphology can also result in the enhancement of the geometric chiral effect, leading to increased magnitude of the second ***g***-factor peak. For example, in Fig. 4b the geometric ***g***-factor of larger 271 nm HgS nanoparticle is 0.0142, which is one order larger than 0.0013 of smaller 30 nm HgS. It is worth noting that while the first ***g***-factor peak at 540 nm is assigned to the crystallographic chirality and its wavelength is independent of size, the increase and red-shifting of the geometric chiral effect for larger sized nanostructures can modify the envelop of CD response induced by the crystallographic handedness. This can explain the reduced magnitude of the first ***g***-factor for larger sized nanostructures, as shown in Fig. 4b. To corroborate our experimental understanding of the chirality interplay between the crystallographic and geometric effects, we have computed CD spectra of all α-HgS nanostructures in Fig. 2 that possess both chiral lattice and chiral morphology, given the structural parameters determined from our sample characterization (Supplementary Table 3). By comparing the computed CD and extinction spectra with experimental results (Supplementary Fig. 16), our model can fully reproduce the observed chiroptical characteristics of nanostructures by incorporating both crystallographic and geometric chirality, further verifying our understanding of the chirality interplay at different length scales.

Importantly, our synthetic scheme in Fig. 2a can allow the flexible combination of crystallographic and geometric handedness, which can offer rich tunability of the chiroptical response in well-defined inorganic nanostructures, mimicking enantiomers and diastereomers in organics and bioorganics. For example, Fig. 4c,d present experimental and computed CD spectra, respectively, of four different combinations of crystallographic and geometric chirality in a twisted triangular bipyramid (with same averaged length of 85 nm): (+)_*C*_-M, (+)_*C*_-P, (−)_*C*_-M and (−)_*C*_-P. We have chosen this medium-size HgS nanostructure as an example to highlight the unique opportunity to finely tailor the nanoscale chiroptical response through the structural engineering shown in Fig. 2a. For this medium-size HgS nanostructure, the variation of chiral morphology mainly modulates the CD feature at shorter wavelengths. From the structural point of view, when comparing among these four epitaxially grown HgS nanostructures, only (+)_*C*_-M/(−)_*C*_-P and (+)_*C*_-P/(−)_*C*_-M represent two pairs of enantiomers that possess opposite handedness of both lattice and morphology, with a totally mirrored CD spectra, and the rest of the pairs are analogous to diastereomeric chiral molecules with non-mirrored CD characteristics. This observation again is consistent with the chiral effects of crystallographic lattice and geometric morphology elucidated in Fig. 4a,b. This result highlights our unique synthetic capability to enable fine tailoring of the chiroptical response at the nanoscale as well as theoretical modelling for describing and predicting chiral phenomena in inorganic nanostructures. By combining a chiral lattice with a chiral morphology, the desired chiroptical response of inorganic nanostructures should be readily achieved.

## Discussion

Both our experimental and theoretical results have demonstrated that the cooperative chiroptical properties of an inorganic nanostructure can be achieved through finely tailored interplay between crystallographic and geometric chirality. In particular, we have developed a solution phase synthetic scheme that can allow the versatile combination of these two different types of handedness, leading to precise chirality engineering at the nanoscale. While various controls of inorganic nanostructures have been extensively studied and achieved, efficient control of chirality at the nanoscale is limited, therefore our current work represents a significant advance in the structural complexity and functionality of inorganic building blocks. Our study opens up various research fronts, ranging from nature-mimicking chiral nanostructures to a wide range of enabled fundamental explorations and technology applications. First, while we have employed cinnabar HgS combined with chiral *D*- and *L*- penicillamine molecules in epitaxial synthesis as an example to demonstrate our synthetic scheme and its sophisticated control of nanoscale cooperative chirality, our methodology illustrated in Fig. 2a is versatile and should be readily extended to other inorganic chiral materials as well as interacting chiral molecules to allow more dynamic modulation of cooperative chirality. For example, there exist a series of chiral molecules that can effectively interact with metal ions, which might lead to different chiral morphologies with different twisting angles (Fig. 1c). To that, two examples by utilizing different chiral molecules and different seed nanoparticles in epitaxial growth are further provided in Supplementary Figs 17 and 18, respectively, to highlight the synthetic opportunity to allow maximum control of chiral structures. Second, chiroptical properties of inorganic materials are particularly important because they are often associated with other phenomena, including spin-selective chemistry and interactions^42^,^43^,^44^,^45^. Therefore, the development of new types of inorganic nanostructures possessing unique and tunable crystallographic and geometric chirality might provide test beds for understanding and controlling spin-dependent or topological phenomena. Indeed, recent works on spin selection and spin transport through chiral biomolecules have led to additional insights^46^,^47^,^48^,^49^. Third, the colloidal chiral nanostructures achieved in the current work can be used as building blocks for hierarchical assembly of mesoscopic structures and devices. This might enable innovative chiral device concepts, for example, by using superchiral fields^50^, and further open up alternative avenues to understand and control nanoscale enantioselective interaction that is currently only observed for bio- and organic-chiral molecules, or to integrate other functional nanoparticles such as plasmonic metal nanoparticles to enable potential fundamental coupling and synergistic functionality^51^,^52^. Last but not least, our theoretical model represents significant theoretical effort to integrate contributions of both crystallographic chirality and geometric chirality of an inorganic nanostructure. The excellent agreement between experimental and theoretical results in our current work not only validates our chiral modelling of inorganic nanostructures but also offer important design guidelines for nanostructures with desired chiroptical properties.

## Methods

### Synthesis of α-HgS seeds for epitaxial growth

All chemicals for this synthesis were used as received without further purification and the purity of water is available in the Supplementary Methods. In a typical synthesis of seed nanoparticles, 64 mg of Hg(NO_3_)_2_˙H_2_O was added into 10 ml of water, followed by injection of 2 ml of penicillamine (either *D*- or *L*-form) aqueous solution (0.09 M) under stirring to get a colourless solution. An aliquot of 0.3 ml of NaOH aqueous solution (2 M) was added to tune the pH value of solution. After that, 1 ml of thioacetamide solution (0.18 M) was added into above mixture solution. The vial was sealed and transferred to a 38 °C water bath. The reaction continued for 15 h under stirring. After reaction, as-prepared seed nanoparticles was separated by mixing with isopropanol (1:6 volume ratio), followed by centrifugation at 5,000 r.p.m. for 10 min. The precipitate was re-dispersed in 10 ml of water.

### Synthesis of α-HgS with chiral twisted bipyramid morphology

All chemicals for this synthesis were used as received without further purification and the purity of water is available in the Supplementary Methods. Synthesis of HgS nanostructures with twisted bipyramid morphology is based on epitaxial growth process, involving preparation of seed nanoparticles, Hg and S precursors. All epitaxial syntheses used either (+)_*C*_ or (−)_*C*_ seeds and were performed by following recipe: in a three-neck round-bottom flask, 0.5 ml of HgS seeds colloidal solution was dispersed in water. The total volume was kept as 2.5 ml. Later, 1 ml of penicillamine (either *D*- or *L*-form) solution (0.09 M) and 0.3 ml of NaOH solution (2 M) were added in sequence. A colourless Hg precursor solution was prepared by dissolving 62 mg of Hg(NO_3_)_2_˙H_2_O in 6 ml of water, followed by addition of 1.8 ml of penicillamine aqueous solution (0.09 M). The resulting solution was stirred for 3 min, and then 0.3 ml of NaOH solution (2 M) was added. The S precursor solution was prepared by dissolving 75 mg of thioacetamide in 10 ml of H_2_O. In a typical epitaxial growth, the seed solution was stirred under a flow of N_2_ gas. The Hg precursor and S precursor solutions were slowly co-injected into the seed solution by using syringe pumps (KDS 220). After reaction, the orange colloidal solution was mixed with isopropanol with volume ratio of 1:4, followed by centrifugation at 4,500 r.p.m. for 10 min. The size of final products was controlled by varying amount of seed nanoparticles, Hg and S precursors with different injection time, with summary of detailed condition provided in Supplementary Table 2.

### Synthesis of α-HgS with achiral nanoellipsoid morphology

All chemicals for this synthesis were used as received without further purification and the purity of water is available in the Supplementary Methods. In a 10-ml glass vial, a colourless precursor solution was prepared by adding 32 mg of Hg(NO_3_)_2_˙H_2_O into 5 ml of water, followed by injection of 1 ml of penicillamine (*D*- or *L*-form) aqueous solution (0.09 M). The resulting solution was stirred for 3 min, and then 0.15 ml of NaOH solution (2 M) was added. After further stirring for 1 min, 1 ml of thioacetamide solution (0.09 M) was added quickly. The vial was then sealed and stirred at 47 °C for 18 h. After reaction, orange colloidal solution was mixed with isopropanol with volume ratio of 1:6, followed by centrifugation at 4,500 r.p.m. for 10 min. The precipitate was re-dispersed in 5 ml of water.

### Synthesis of α-HgS with achiral nanocube morphology

All chemicals for this synthesis were used as received without further purification and the purity of water is available in the Supplementary Methods. An aliquot of 32 mg of Hg(NO_3_)_2_˙H_2_O was added to 5 ml of H_2_O, followed by injection of 1 ml of *D*-penicillamine (or *L*-penicillamine) aqueous solution (0.09 M). After that, the pH was tuned by addition of 0.15 ml of NaOH solution (2 M). After further stirring for 1 min, 0.9 ml of thioacetamide solution (0.3 M) was injected quickly. The vial was then sealed and stirred at 60 °C. The α-HgS nanoparticles with cubic morphology were obtained after 18 h of reaction.

### Synthesis of α-HgS with achiral nanorod morphology

All chemicals for this synthesis were used as received without further purification and the purity of water is available in the Supplementary Methods. The nanorods were synthesized using a hydrothermal method. In a typical synthesis, 32 mg of Hg(NO_3_)_2_˙H_2_O was dissolved in 4 ml of H_2_O in a 10-ml Teflon-lined autoclave, followed by addition of 1 ml of *D*-penicillamine aqueous solution (0.09 M) and 0.145 ml of NaOH solution (2 M). After further stirring for 1 min, 1 ml of thioacetamide solution (0.09 M) was injected quickly. The autoclave was sealed and heated at 72 °C for 40 h in an oven that was preheated to the desired temperature, and then was cooled down to room temperature. The product was collected by centrifugation with isopropanol.

### Synthesis of α-HgS with achiral nanowire morphology

All chemicals for this synthesis were used as received without further purification and the purity of water is available in the Supplementary Methods. The nanowires were synthesized using a hydrothermal method. In a glass vial, 16 mg of Hg(NO_3_)_2_˙H_2_O was dissolved in 5 ml of H_2_O, followed by addition of 0.45 ml of *D*-penicillamine aqueous solution (0.1 M). Then 0.075 ml of NaOH solution (2 M) was added. After stirring for 1 min, 1.38 ml of above solution was added into 4.38 ml of H_2_O in a 10-ml Teflon-lined autoclave. An aliquot of 0.113 ml of thioacetamide solution (0.1 M) was added under stirring. After further stirring for 3 min, the autoclave was sealed and heated in an oven with preset temperature of 110 °C. After reaction for 6 h, the autoclave was removed from the oven and cooled to room temperature by directly flushing with cold water. The product was collected by centrifugation with isopropanol. Nanowires with different aspect ratio were achieved by varying concentration of precursor and reaction time.

### Modelling and computation of CD and extinction spectra

Detailed description of electromagnetic modelling is provided in Supplementary Note 1. All structural 3D models were built by using Solidworks. Simulation was completed in the frequency domain by using RF module of COMSOL FEM package (5.2) with structures imported from Solidworks. Simulations in Fig. 1c were performed with single incident wave vector of 30° relative to the *c* axis of HgS nanoparticle. All other computed CD and absorption spectra for comparing with ensemble experimental data were performed by averaging incident wave vectors around the chiral nanoparticle.

### Data availability

The data that support the findings of this study are available from the corresponding author on request.

## Additional information

**How to cite this article:** Wang, P.-P. *et al*. Cooperative expression of atomic chirality in inorganic nanostructures. *Nat. Commun.* **8,** 14312 doi: 10.1038/ncomms14312 (2017).

**Publisher's note:** Springer Nature remains neutral with regard to jurisdictional claims in published maps and institutional affiliations.

## Supplementary Material

Supplementary Information Supplementary Figures, Supplementary Tables, Supplementary Note, Supplementary Methods and Supplementary References.

Supplementary Movie 1 Side view of 3D reconstruction of a twisted triangular bipyramid HgS nanocrystal. A movie shows the 3D electron tomography reconstruction of a single nanocrystal, confirming assignment of geometric morphology.

Supplementary Movie 2 Top view of 3D reconstruction of a twisted triangular bipyramid HgS nanocrystal. A movie shows the 3D electron tomography reconstruction of a single nanocrystal, confirming its three-fold structural symmetry.
