# Supplementary material for: Cooperative expression of atomic chirality in inorganic nanostructures
Source: Nat Commun. 2017 Feb 2;8:14312. doi: 10.1038/ncomms14312 (PMC5296657; doi:10.1038/ncomms14312)
Supplement: Supplementary Information — Supplementary Figures, Supplementary Tables, Supplementary Note, Supplementary Methods and Supplementary References. [file ncomms14312-s1.pdf]

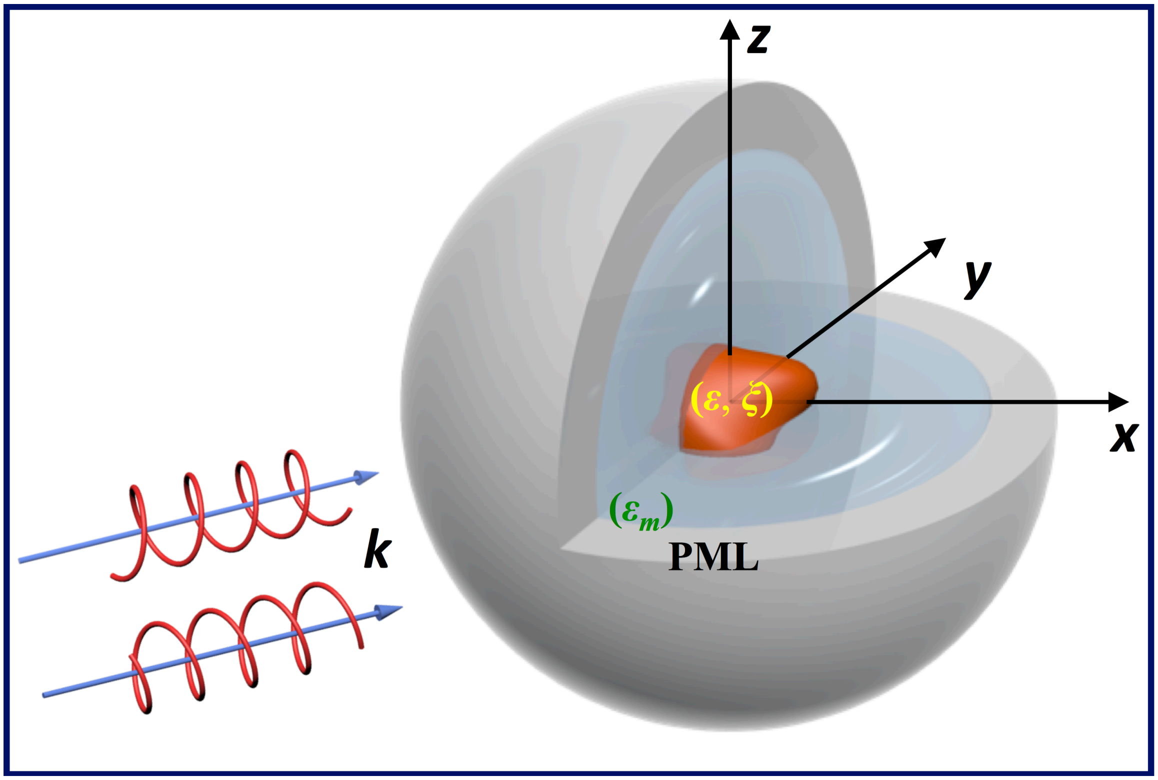

Supplementary Figure 1 | Electromagnetic core-shell model for computing chiroptical response of a chiral nanostructure with arbitrary morphology. A chiral nanostructure with arbitrary morphology and parameters of (ε, ξ) is placed in the origin as core. The chiral nanostructure is encapsulated by an achiral spherical shell with dielectric constant of ε_m, whose size is much bigger than chiral core to simulate the environmental medium. Since our circular dichroism (CD) measurement is always performed in an achiral solvent (such as water), chiral parameter ξ_m of media is always set to be zero. A perfectly matching layer (PML) boundary condition is applied to the outermost surface of environmental shell in a finite element method (FEM) simulation. Optical response of chiral nanostructure in the model is computed under illumination of left and right- circularly polarized plane waves with incident wave vector $\boldsymbol{k}$. In order to compare with our experiment, computed CD spectra are averaged over all $\boldsymbol{k}$ directions. See also Supplementary Note 1 for detailed discussion.

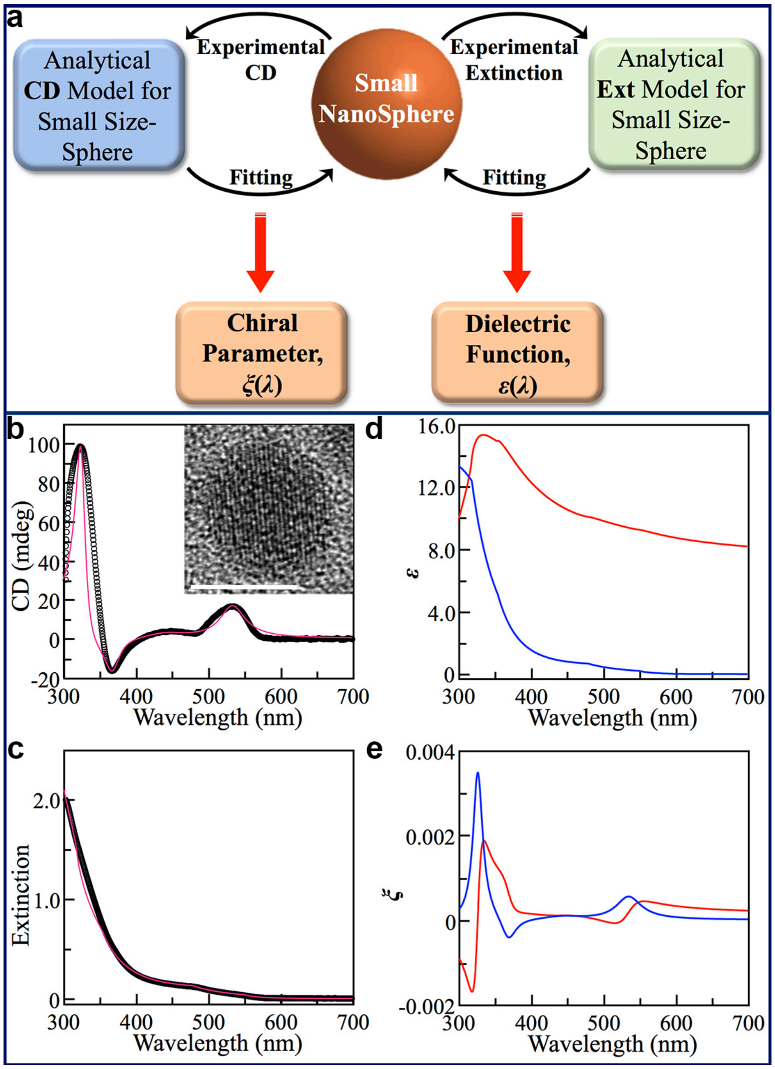

Supplementary Figure 2 | Parametric modeling of materials parameters (ε, ξ) of a chiral nanostructure. a, Schematic of parametric modeling. The key is to acquire CD and absorption spectra of small sized spherical chiral nanoparticles and fit with corresponding analytical solution to extract parameters (ε, ξ). **b** and **c**, Experimental (black sphere) and fit (pink curve) spectra of CD and absorption of 12 nm- sized HgS nanoparticles, respectively. (**Inset**) of **b**, A typical transmission electron microscopy (TEM) image of a single α-HgS nanoparticle for acquiring experimental data, showing quasi- spherical morphology. Scale bar, 10 nm. **d** and **e**, Imaginary (blue) and real (red) parts of parameters ε and ξ of α-HgS, respectively, which are obtained based on fits in **b** and **c**. See also Supplementary Note 1 for detailed discussion.

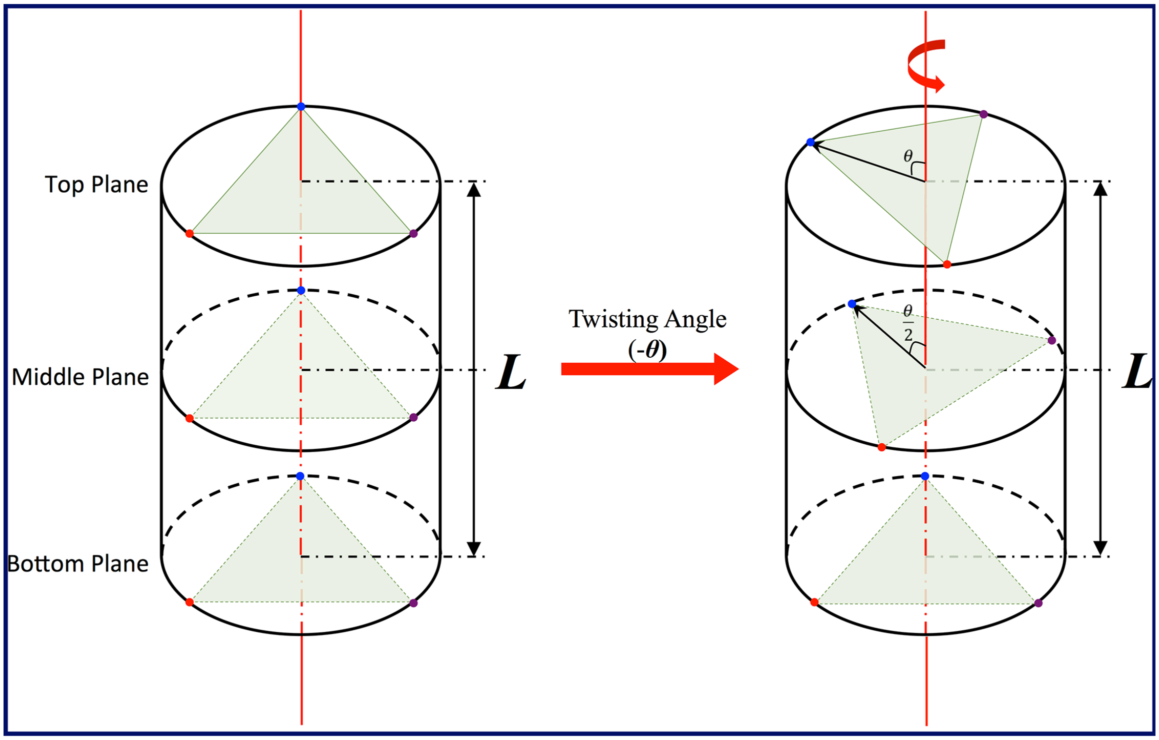

Supplementary Figure 3 | Structural model of twisting. Cylindrical plot of a twisted structure is applied to illustrate definition of twisting and twisting angle, θ in structural modeling. The plot shows a right-handed twist (resulting in P morphology). The twisting angle of a selected cross-section plane along axis is proportional to the distance between this plane and the bottom plane, d, as $\theta_d = -\left(\frac{d}{L}\right)\theta$. For a left-handed twist (resulting in M morphology), the twisting angle is defined to be positive.

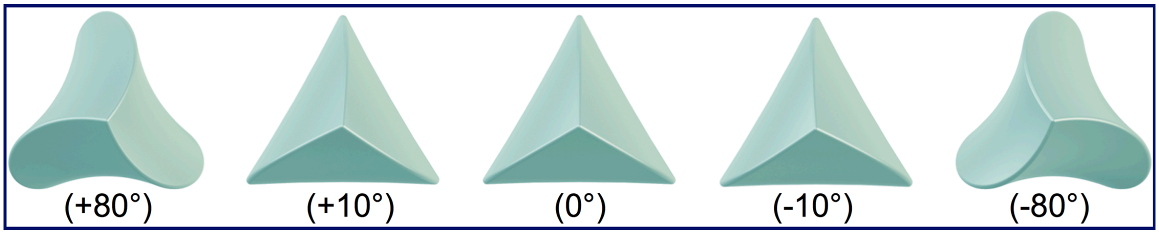

Supplementary Figure 4 | Top- view of triangular bipyramid structures with different twisting $\boldsymbol{\theta}$. These top-view models correspond to structures presented in Fig. 1**c**.

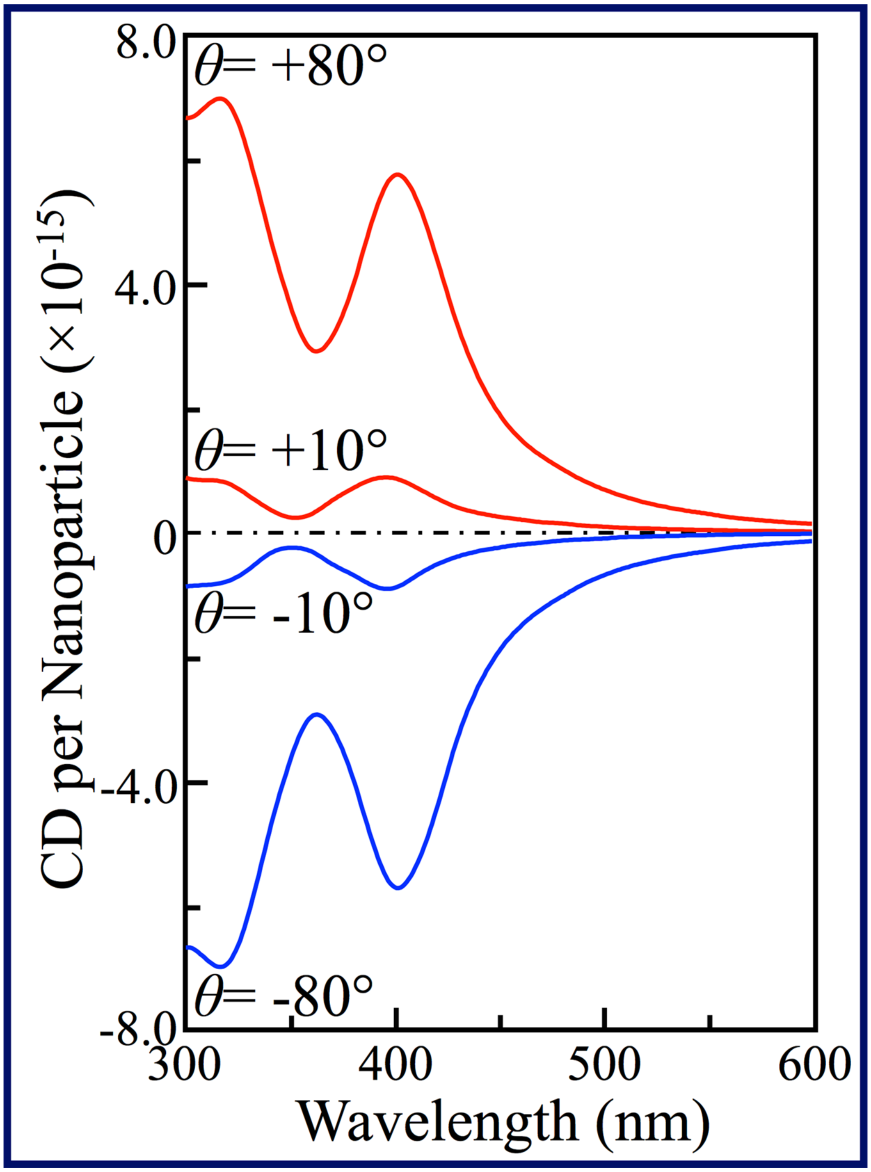

Supplementary Figure 5 | Computed contribution of CD response merely from chiral morphology. In order to evaluate contribution of CD response from morphological effect in Fig. 1**c**, CD spectra are computed by setting chiral parameter ξ to be zero, and all other parameters remain the same as those for Fig. 1**c**.

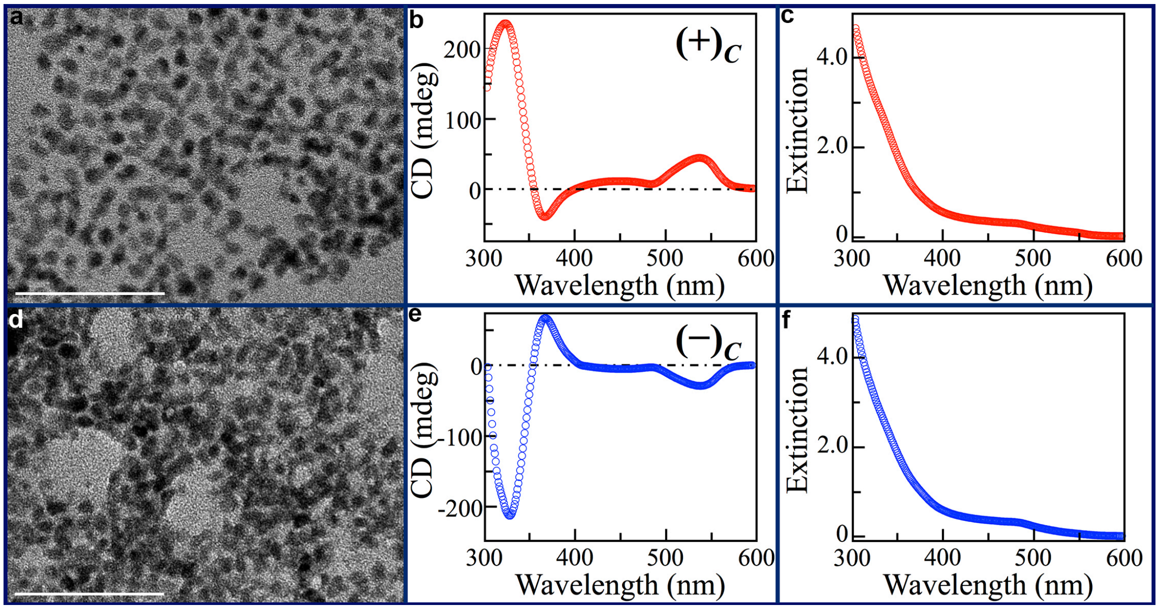

Supplementary Figure 6 | Seed α-Hg nanoparticles possessing crystallographic handedness. **a**, **b** and **c**, Typical large- scale TEM image, CD spectroscopy and extinction spectroscopy of (+)$_C$- seed nanoparticles, respectively. Scale bar, 100 nm. **d**, **e** and **f**, Typical large scale TEM image, CD spectroscopy and extinction spectroscopy of (−)$_C$- seed nanoparticles, respectively. Scale bar, 100 nm.

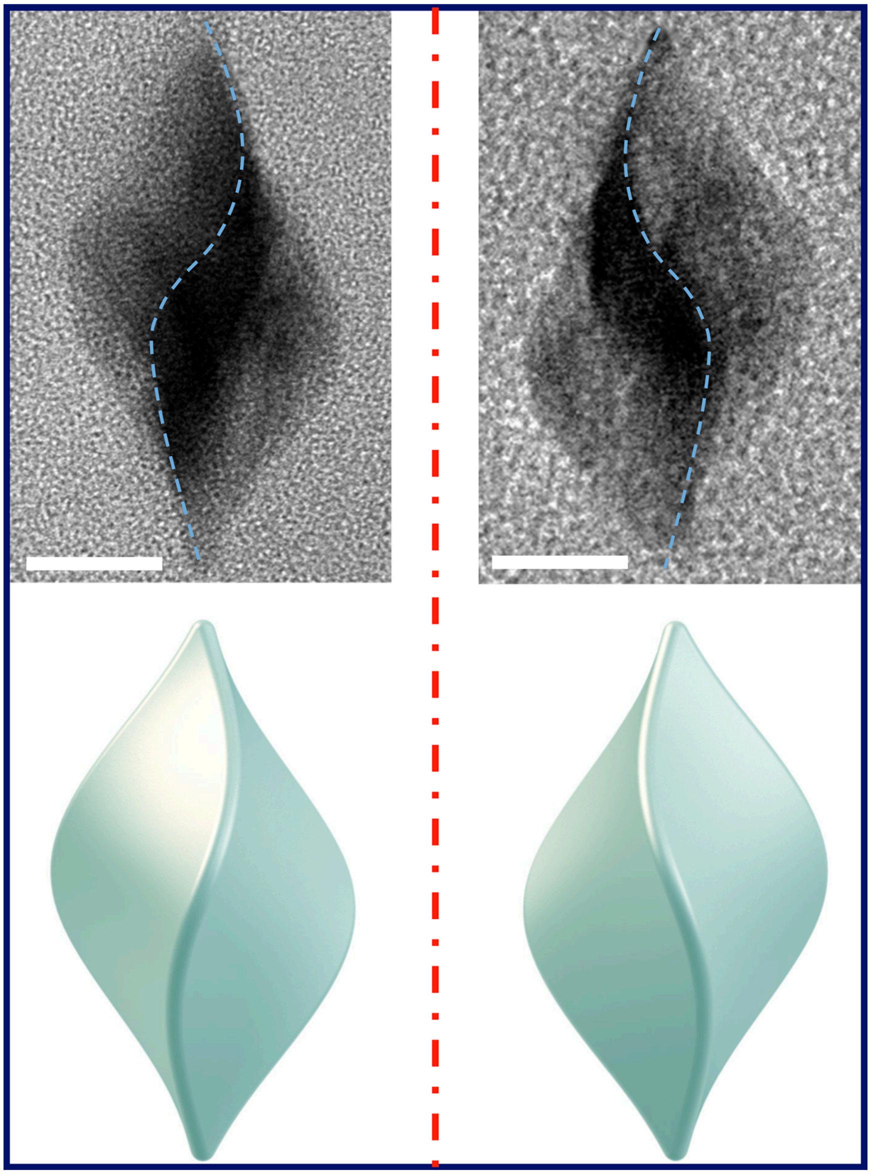

Supplementary Figure 7 | Structural models of two mirrored α-HgS nanostructures in Figs. 2d and 2e. TEM images are the same as presented in Figs. 2**d** and **2e**, respectively. Scale bar, 30 nm.

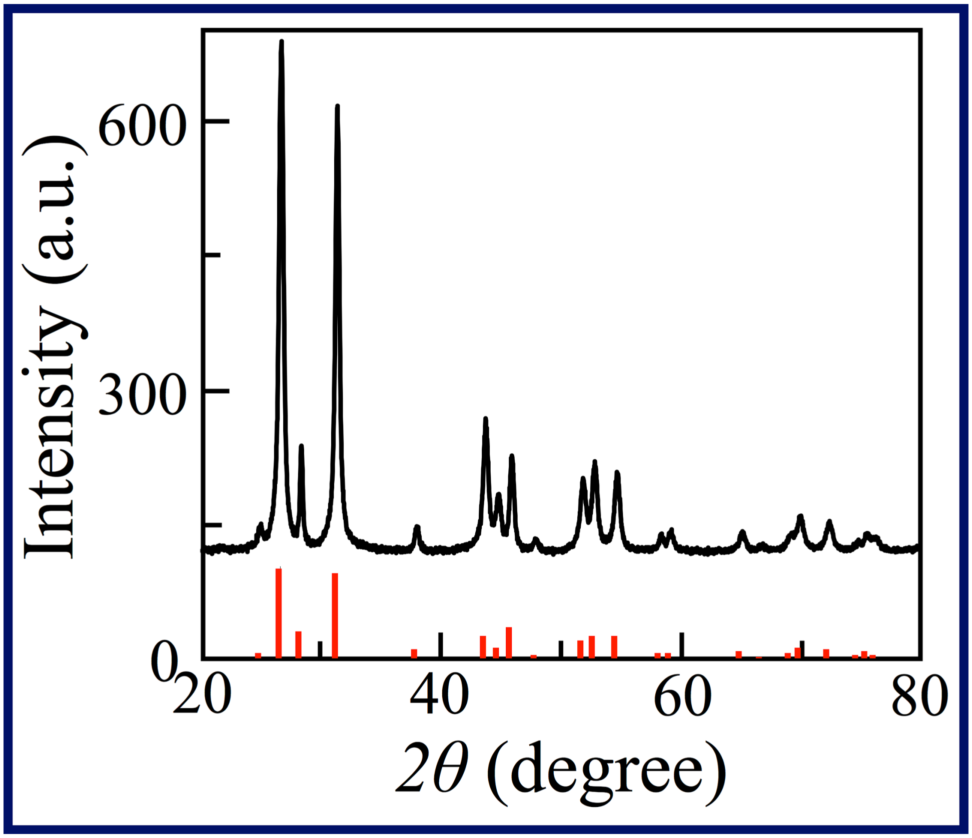

Supplementary Figure 8 | X-ray diffraction (XRD) characterization of α-HgS nanoparticles with chiral morphology. The experimental data was acquired from sample in Fig. 1**b**. For comparison and assignment purpose, standard XRD peaks of cinnabar HgS are also presented (vertical red lines, JCPDS# 06-0256). Excellent agreement between experimental data and database confirms our assignment of cinnabar lattice of as-synthesized HgS nanoparticles with chiral morphology by following the synthetic scheme in Fig. 2**a**.

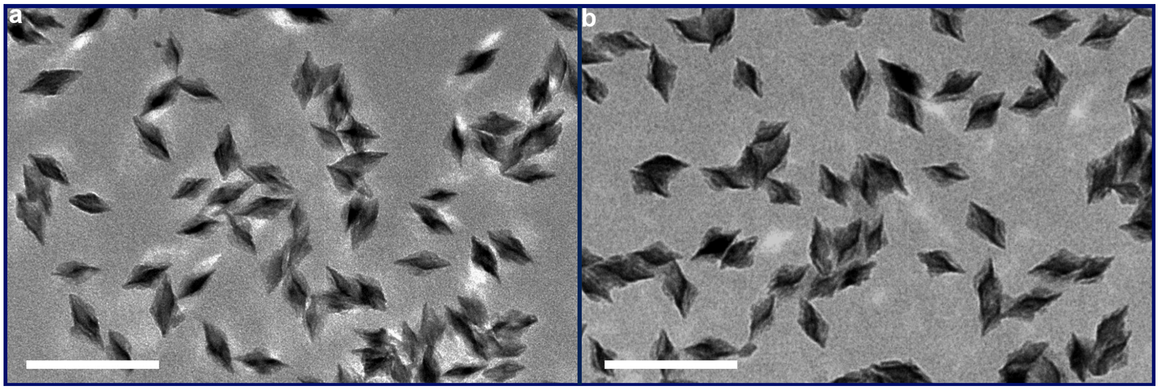

Supplementary Figure 9 | The twisted triangular bipyramid α-HgS nanoparticles synthesized from (−)$_C$- seed nanoparticles. a and **b**, Typical TEM image of as-synthesized 76.9 nm- long HgS nanoparticles with morphological handedness of M and P, respectively. As compared with samples in Fig.2**b** and 2**c**, these nanoparticles are synthesized from (−)$_C$- seed nanoparticles. Scale bar, 200 nm.

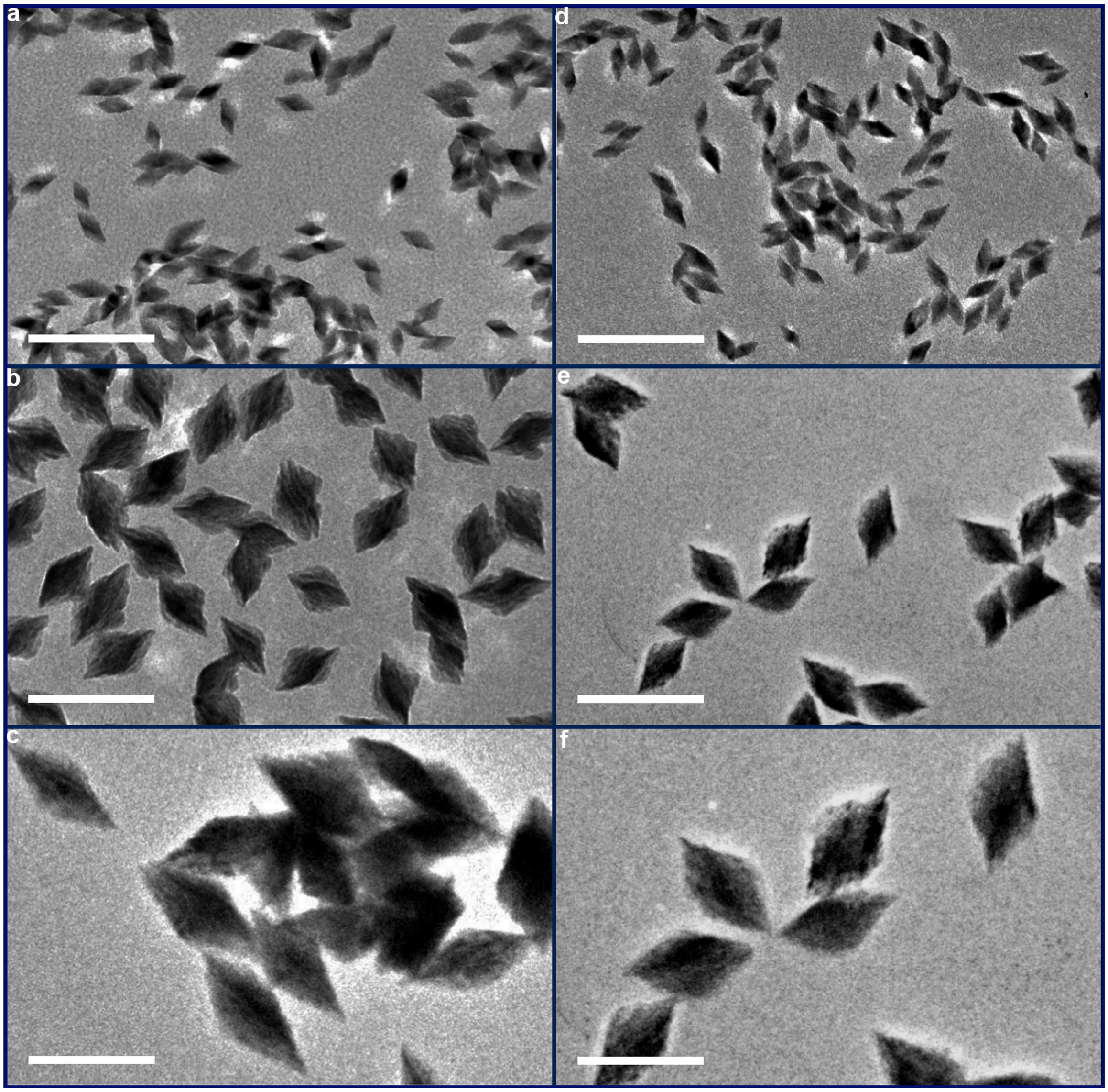

Supplementary Figure 10 | Large- scale TEM images of samples presented in Fig. 2f. a-c, Typical TEM images of $(+)_C$-M HgS nanoparticles with averaged length of 54 nm, 145 nm, and 201 nm, respectively. Scale bar, 200 nm. **d-f**, Typical TEM images of $(+)_C$-P HgS nanoparticles with averaged length of 54 nm, 145 nm, and 201 nm, respectively. Scale bar, 200 nm.

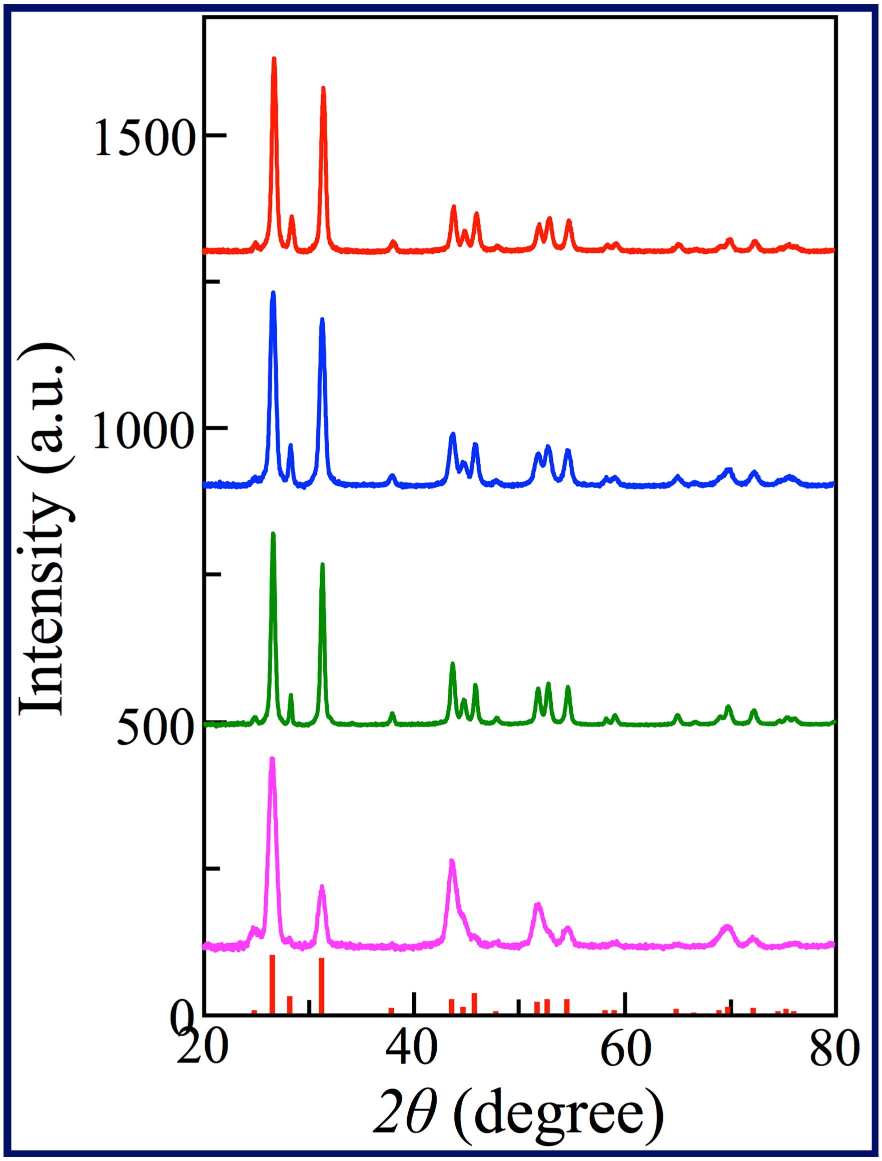

Supplementary Figure 11 | XRD characterization of HgS nanoparticles with different achiral morphologies. Red, blue, green and pink curves are experimental data for nanocubes (Fig. 3**a**), nanoellipsoids (Fig. 3**b**), nanorods (Fig.3**c**) and nanowires (Fig. 3**d**), respectively. For comparison and assignment purpose, standard XRD peaks of cinnabar HgS are also presented (vertical red lines, JCPDS# 06-0256). Excellent agreement between experimental data and database confirms our assignment of cinnabar lattice of all as-synthesized HgS nanoparticles with achiral morphology.

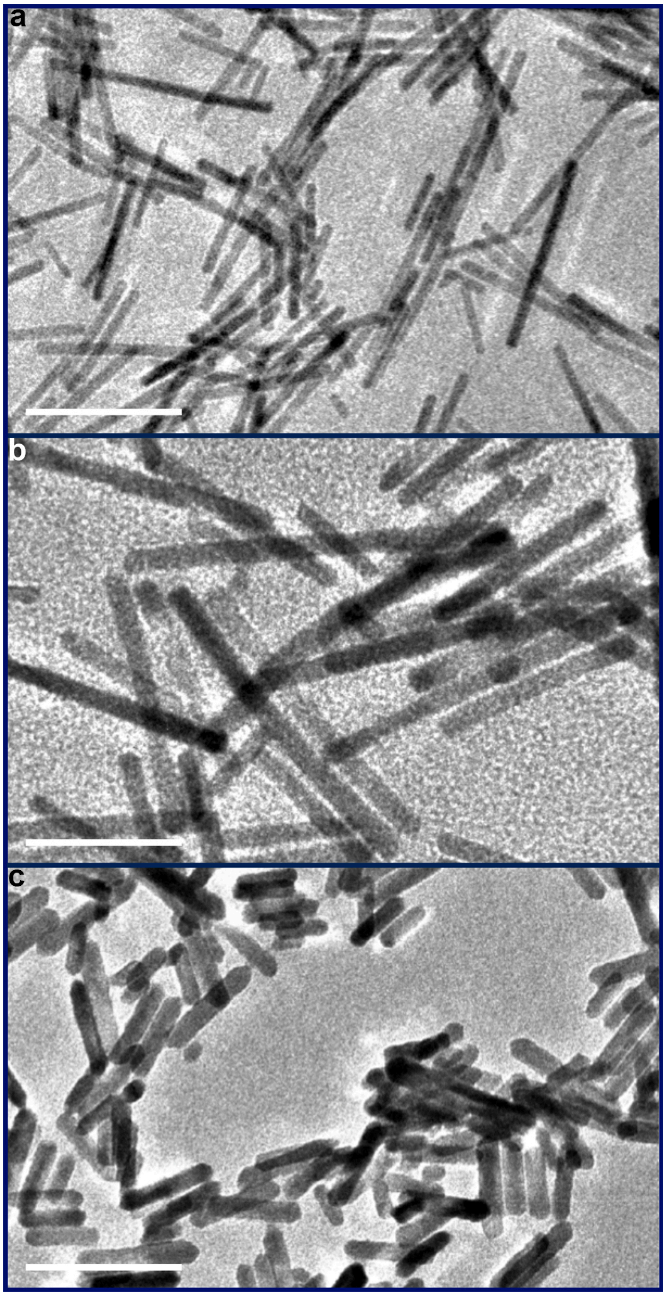

Supplementary Figure 12 | Typical TEM images of α-HgS nanowires ($(+)_C$) with different aspect ratios. a, 110 nm long nanowires with 10 nm diameter (denoted as "Nanowires 2" in Fig. 4a). **b**, 110 nm long nanowires with 18 nm diameter (denoted as "Nanowires 3" in Fig. 4a). **c**, 55 nm long nanowires with 18 nm diameter (denoted as "Nanowires 4"). Scale bar, 100 nm.

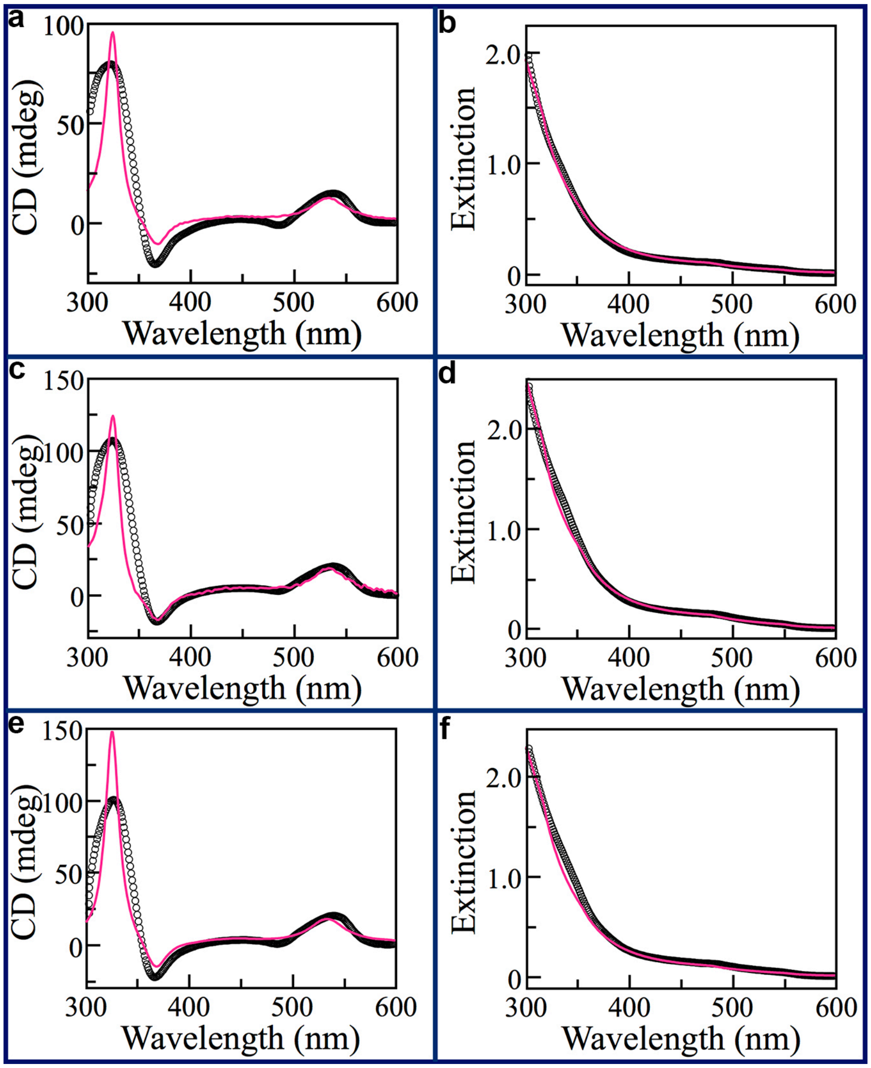

Supplementary Figure 13 | Comparison of experimental and computed CD and extinction spectra of (+)$_C$ HgS nanoparticles with different achiral morphologies. (**a**, **b**), (**c**, **d**), and (**e**, **f**), The CD and extinction spectra of HgS nanoparticles with nanocubic (Fig. 3**a** in main text), nanoellipsoidal (Fig. 3**d** in main text) and nanorod (Fig. 3**g** in main text) morphology, respectively. Black circles, experimental data. Pink curves, computed spectra.

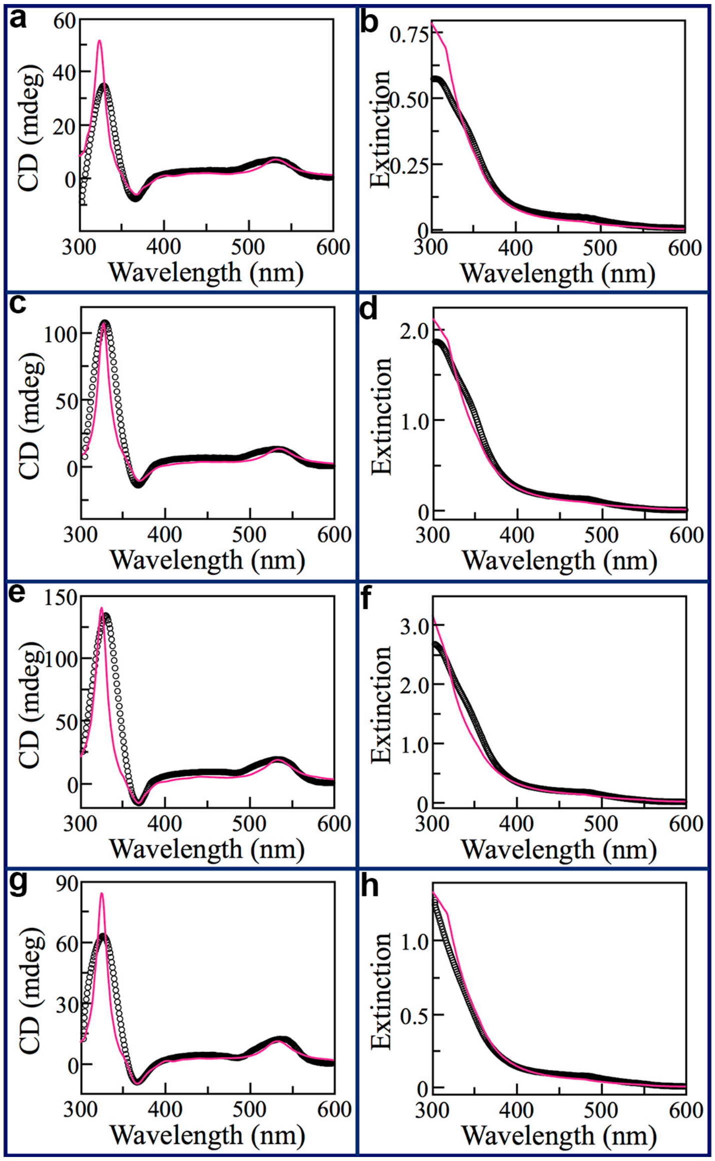

Supplementary Figure 14 | Comparison of experimental and computed CD and extinction spectra of (+)$_C$ HgS nanowires with different aspect ratios. (**a**, **b**), (**c**, **d**), (**e**, **f**) and (**g**, **h**), The CD and absorption spectra of α-HgS nanowires presented in Fig. 3**j**-3**l** in main text (Nanowires 1), Supplementary Fig. 12**a** (Nanowires 2), Supplementary Fig. 12**b** (Nanowires 3), and Supplementary Fig. 12**c** (Nanowires 4), respectively. Black circles, experimental data. Pink curves, computed spectra.

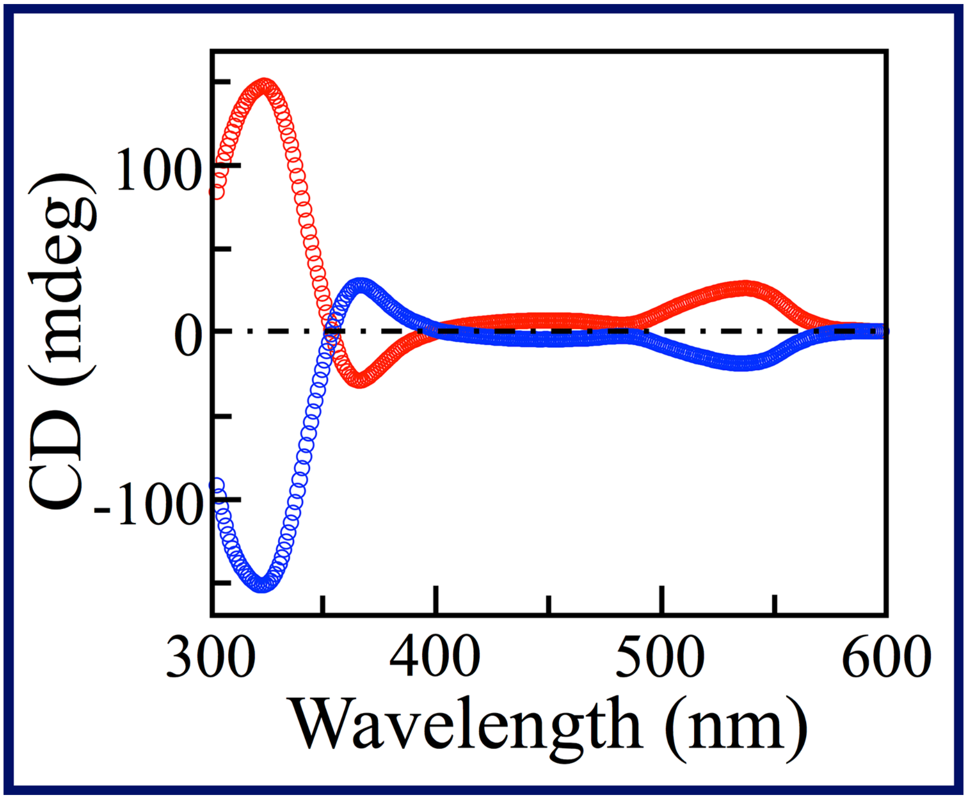

Supplementary Figure 15 | Experimental CD spectra of α-HgS nanocubes possessing different crystallographic handedness. (Red) nanocubes with $(+)_C$-lattice. (Blue) nanocubes with $(-)_C$- lattice.

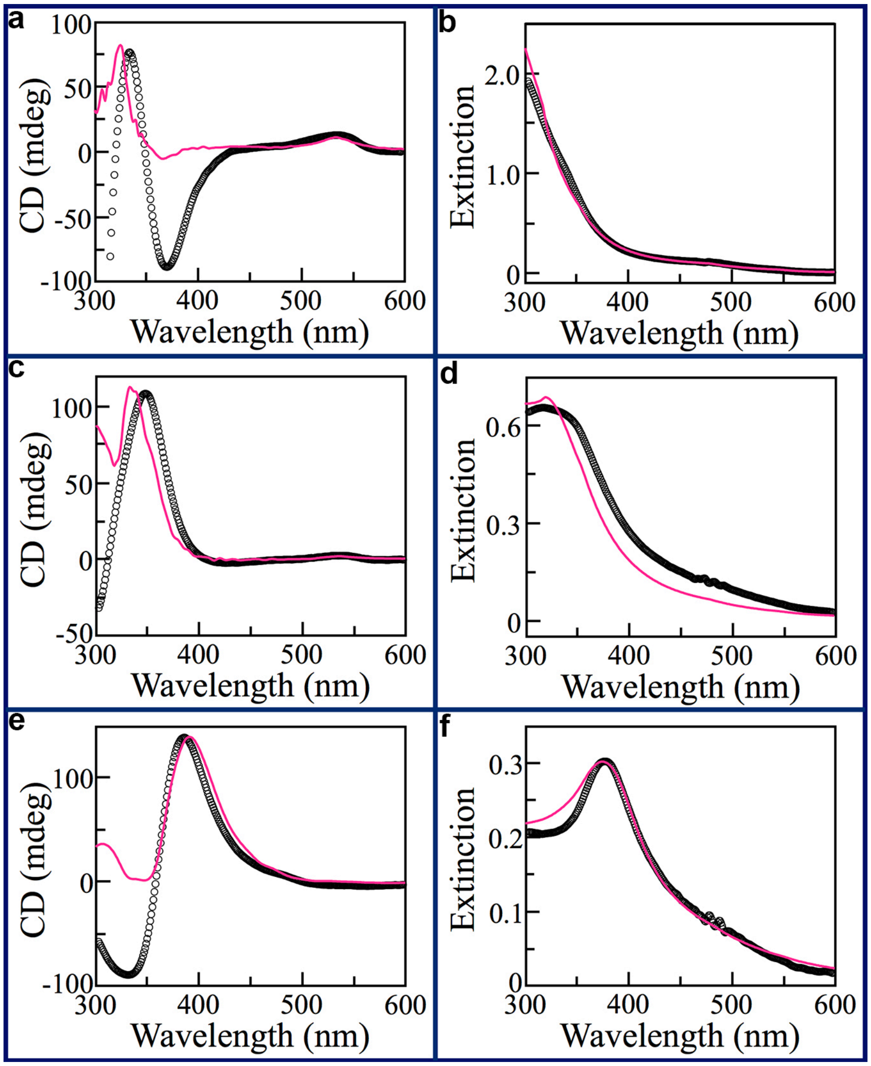

Supplementary Figure 16 | Comparison of experimental and computed CD and extinction spectra of (+)$_C$-M HgS nanoparticles possessing different size. (**a**, **b**), (**c**, **d**) and (**e**, **f**), 54 nm, 145 nm and 201 nm long, respectively. Black circles, experimental data. Pink curves, computed spectra.

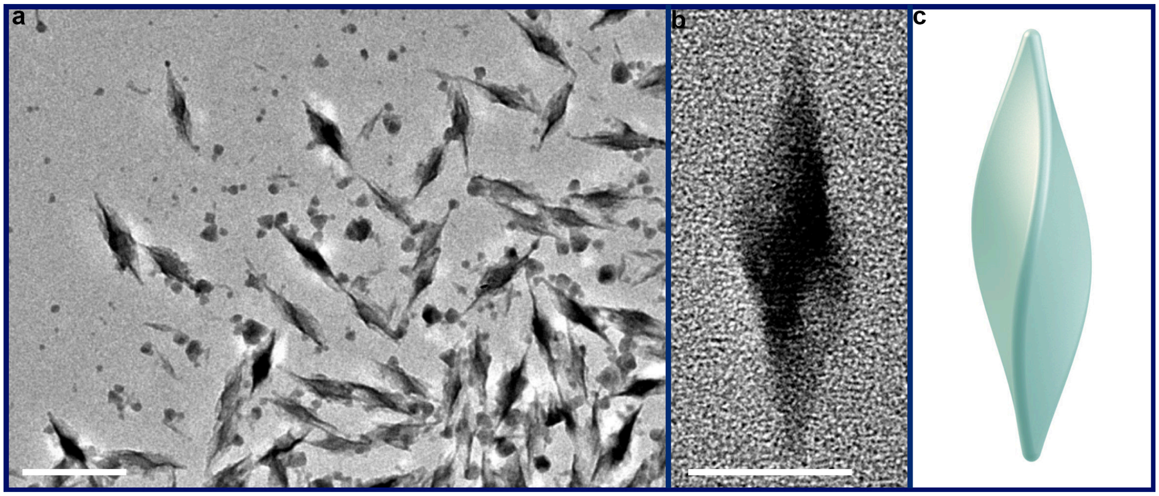

Supplementary Figure 17 | Twisted triangular bipyramid nanostructures with different aspect ratio and twisting angle enabled by using different chiral molecules. **a**, Typical TEM image of sample synthesized by following scheme in Fig. 2**a** but with a different chiral molecule (N-Acetyl-D-penicillamine, CAS 15537-71-0) during the epitaxial synthesis. Scale bar, 100 nm. **b**, Typical TEM image of an individual nanostructure, showing an aspect ratio of 3.4 and twisting angle of 100°. Scale bar, 30 nm. **c**, Structural model built to compare with the image in **b**.

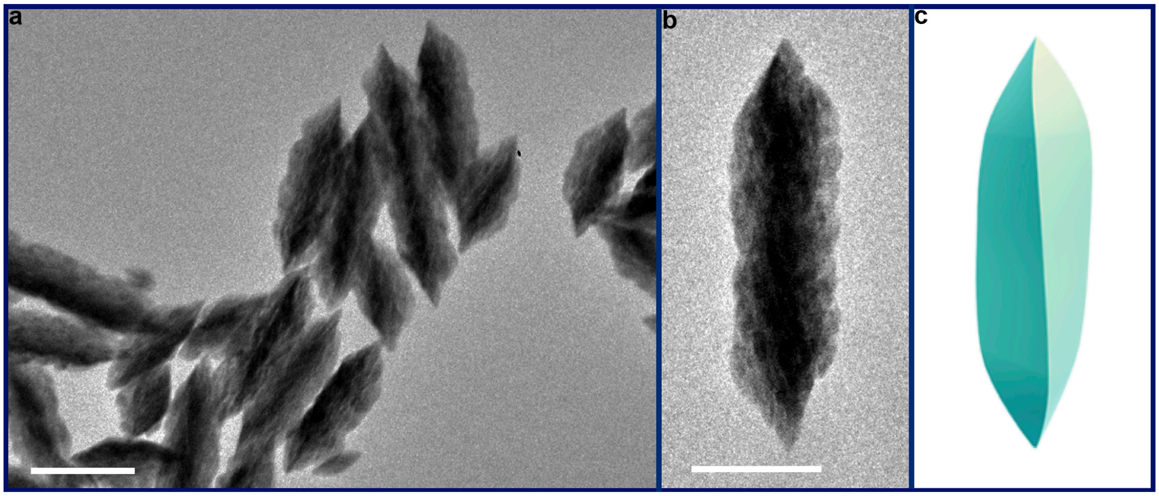

Supplementary Figure 18 | Twisted triangular bipyramid nanostructures with different aspect ratio and twisting angle enabled by using nanorods as seed nanoparticles. a, Typical TEM image of sample synthesized by following scheme in Fig. 2**a** but with nanorods as seed nanoparticles for epitaxial synthesis. Scale bar, 200 nm. **b**, Typical TEM image of an individual nanostructure, showing an aspect ratio of 3.9 and twisting angle of 25°. Scale bar, 100 nm. **c**, Structural model built to compare with the image in **b**.

**Fitting Parameters for ε**			**Fitting Parameters for ξ**			
$\boldsymbol{i}$	e_{ti}/eV	e_{ui}/eV	$\boldsymbol{i}$	e_i/eV	Γ_i/e_i	$a_i/\left(10^{-5}/\Gamma_i\right)$
**1**	3.9	0.4	**1**	2.32	0.04	-0.467
**2**	3.5	0.26	**2**	2.8	0.08	-0.4
**3**	2.59	0.25	**3**	3.37	0.035	0.8
**4**	2.26	0.08	**4**	3.81	0.025	-3.2

Supplementary Table 1 | Optimized parameters of $\left(\xi,\ \varepsilon\right)$ of α-HgS. See also Supplementary Note 1.

Structure	Averaged Length (nm)	Seed Volume (ml)	Hg Precursor (ml)	S Precursor (ml)	Reaction Time (hrs)
**1**	30.9	0.5	1	0.275	0.5
**2**	53.6	0.5	2	0.55	2
**3**	76.9	0.5	4	1.1	4
**4**	85.5	0.3	4	1.1	4
**5**	144.8	0.1	4	1.1	4
**6**	201.2	0.028	4	1.1	4
**7**	271.2	0.016	4	1.1	4

Supplementary Table 2 | Summary of growth condition for different sizes of α-HgS with twisted triangular bipyramid morphology. Typical synthetic procedure is provided in Methods.

Structure	Length (nm)	Width (nm)	Aspect Ratio	Twisting Angle (°)
**2**	53.6 ± 6.3	26.4 ± 2.5	2.03 ± 0.17	66.7 ± 4.9
**3**	76.9 ± 5.8	40.6 ± 2.7	1.90 ± 0.15	65.3 ± 3.5
**4**	85.5 ± 6.4	41.7 ± 2.9	2.05 ± 0.15	61.0 ± 6.0
**5**	144.8 ± 6.9	74.0 ± 2.6	1.96 ± 0.11	57.0 ± 6.5
**6**	201.2 ± 6.1	102.8 ± 6.1	1.96 ± 0.14	60.3 ± 6.7
**7**	271.2 ± 7.1	141.1 ± 10.2	1.93 ± 0.12	59.5 ± 4.5

Supplementary Table 3 | Summary of statistical analysis of structural parameters of epitaxial grown samples. Samples used for this statistical analysis are $(+)_C$-M. We have also observed that for nanoparticles grown from same seed nanoparticles (possessing either $(+)_C$ or $(-)_C$ lattice), their corresponding P and M morphologies show exactly the same structural parameters other than opposite twisting orientation (see also Figs. 2d, 2e, 2i-2n).

Supplementary Note 1: Electromagnetic Modeling and Simulation of Crystallographic and Morphological Handedness

Optical response of a matter that possesses both crystallographic chirality and morphological chirality can be described by the combination of Maxwell's equations and constitutive relations[1,2]. The Gaussian (CGS) units are applied in our model and computation.

Light-matter interaction involving chiral media

Our core-shell model (see Supplementary Fig. 1) incorporates a crystalline lattice with arbitrary geometric shape (that can be either chiral or achiral) as core particle, which is embedded in a spherical non-chiral dielectric environmental matrix shell. The dimension of non-chiral dielectric matrix shell is typically much larger than that of central nanostructure to account for the environmental media in a CD measurement. In general, the interaction between electromagnetic wave (such as light) and matter is describable in terms of a set of parameters, $\left(\xi,\ \varepsilon,\ \mu\right)$ that relate the fields $\vec{D}$ to $\vec{E}$ and $\vec{B}$ to $\vec{H}$ via the constitutive relations, where ξ is chiral parameter that quantifies chiral asymmetry (the ξ is related to rotation of the plane of polarization when monochromatic electromagnetic wave passes through chiral media, such as chiral atomic lattice), ε is dielectric function, and μ is relative permeability. The system is excited by an external monochromatic

electromagnetic wave with frequency ω, in which the electric and magnetic field components, $\overline{E}$ and $\overline{H}$ at location $\overline{r}$ have a time dependence given by the factor of $\exp(-i\omega t)$:

$$\begin{cases} \overline{E} = \overline{E}_0 e^{i(\overline{k}\bullet\overline{r}-\omega t)} \\ \overline{H} = \overline{H}_0 e^{i(\overline{k}\bullet\overline{r}-\omega t)} \end{cases} \tag{1}$$

where $\overline{k}$ is incident wave vector, $\left|\overline{E}_0\right|$ and $\left|\overline{H}_0\right|$ are amplitudes of electric and magnetic fields, respectively. To perform a FEM simulation, a PML is also added in our model as the outermost shell to absorb launched waves at the boundary of dielectric medium, which simulates an infinite environment and helps truncate computational domains.

In our current model, we treat all our nanostructures as isotropic non-magnetic chiral media, in which the constitutive equations can be given by[3,4]:

$$\begin{cases} \overline{D} = \varepsilon_{BPE}\overline{E} + i\xi\overline{B} \\ \overline{H} = \mu_{BPE}^{-1}\overline{B} + i\xi\overline{E} \end{cases} \tag{2}$$

where ε_{BPE} is the Bassiri-Papas-Engheta dielectric function and is related to $\varepsilon_{BPE} = \varepsilon - \xi^2\mu$, and $\mu_{BPE} = \mu$. In our model, we therefore adopt above constitutive relations with ε and μ independent of the chiral parameter ξ. This can allow us to evaluate chirality originating from crystallographic and geometric effects independently. Furthermore, we consider only isotropic chiral lattice in our current work, that is, the ξ is isotropic. While in principle the ξ should be anisotropic depending on symmetry group

of a lattice, as a first order approximation, assumption of isotropy can simplify calculation as well as determination of parameters, and allow us to address underlying basic physics, but in the future, more accurate description of anisotropic ξ should further improve the agreement between experiment and simulation.

Since materials of interest in our current study are non-magnetic, we can have μ for both chiral media and dielectric matrix as the constant of 1 (refs. 4-6). For achiral dielectric matrix surrounding the chiral media, we also have $\xi_m = 0$ and $\varepsilon_m = \sqrt{n_m}$, where n_m is its refractive index. In our computation, the thickness of PML shell is set to be $\lambda / \left(2n_m\right)$ in order to completely absorb launched electromagnetic wave at the boundary of environmental dielectric matrix, where λ is the wavelength of incident electromagnetic wave.

The time-averaged Poynting vector (energy flux density) is given by[1,7]:

$$\left\langle \vec{S} \right\rangle = \frac{1}{2}\mathrm{Re}\left[\vec{E}\left(\vec{r},\omega\right) \times \vec{H}^{*}\left(\vec{r},\omega\right) \right] \tag{3}$$

Thus the energy flux density of scattered field through a given surface with a normal unit vector $\vec{n}$ is given by:

$$\left\langle S_{scat} \right\rangle = \left\langle \vec{S}_{scat} \right\rangle \bullet \vec{n} = \sum_{i=x,y,z} n_i \left\langle S_{i,scat} \right\rangle \tag{4}$$

Therefore, the absorption, scattering and extinction cross sections can be determined by[8]:

$$\sigma_{abs} = \frac{1}{\langle S_0 \rangle} \iiint_V Q dV \tag{5}$$

$$\sigma_{scat} = \frac{1}{\langle S_0 \rangle} \iint_S \langle S_{scat} \rangle dS \tag{6}$$

$$\sigma_{ext} = \sigma_{abs} + \sigma_{scat} \tag{7}$$

where Q is the dissipative loss density, the integral volume V is the whole physical region (by summing volume of environmental matrix and central chiral media), the integral surface S is the interface between physical region and PML, $\langle S_0 \rangle$ is the time- averaged energy flux density of background plane wave that is equal to $\langle S_0 \rangle = \frac{1}{2} \frac{n_m |\vec{E}_0|^2}{Z_0}$, where Z_0 is the characteristic impedance of vacuum.

In order to evaluate frequency- dependent CD response, the extinction cross sections of the chiral media under illumination of left- and right- handed circularly polarized plane waves, σ_{ext}^{+} and σ_{ext}^{-}, are computed independently, and then the CD spectrum can be evaluated by $CD(\omega) = \sigma_{ext}^{+} - \sigma_{ext}^{-}$. Since all optical measurements in current work are ensemble measurements, the CD resulted from all directions of $\vec{k}$ needs to be averaged around the chiral media, when comparing with experimental results.

Parametric modeling of optical function of non-magnetic crystallographic lattice

One of the keys for computation with model described above is to determine parameters set for chiral nanostructures, which might not be always available.

Supplementary Fig. 2a shows a general self- consistent approach to determine $(\xi,\ \varepsilon)$ of a non-magnetic chiral material ($\mu = 1$) from experimental results. This approach is based on the fact that analytical solution of absorption and CD spectrum for nanoparticles with small size and spherical morphology can be available to allow comparison with related experimental results for parameter fitting. For example, for a small sized spherical particle, theoretical absorption cross section can be given by[5,9]:

$$\sigma_{abs} = B\omega \operatorname{Im}\left(\varepsilon_r(\omega)\right)\left|\frac{3\varepsilon_m}{2\varepsilon_m + \varepsilon_r(\omega)}\right|^2 \tag{8}$$

where $B = \dfrac{V}{c\sqrt{\varepsilon_m}}$ is a constant (c is the speed of light and V is the volume of nanoparticle). The dielectric function of chiral nanoparticles ε_r, is a complex number given by $\varepsilon_r = \varepsilon_1 + i\varepsilon_2$. The imaginary part can be described by a modified Tauc-Lorentz model (that is, combination of Tauc joint density of states with the Lorentz oscillator)[10,11]. For only a single inter-band transition is considered:

$$\varepsilon_2 = \begin{cases} \left(\dfrac{e_0}{E}\right) \bullet \dfrac{F(E-e_t)^2\Gamma}{(E^2-e_0^2)^2+\Gamma^2E^2}, & \left(E > e_t\right) \\ \left(\dfrac{C_1}{E}\right) \bullet \exp\left(\dfrac{E-e_t}{e_u}\right), & \left(E \le e_t\right) \end{cases} \tag{9}$$

where e_t is the demarcation energy between the Urbach tail transition and the inter-band transition, e_u is the Urbach width describing absorption tail associated with inter-band

transition, e_0 is the resonant energy of chiral nanoparticles, F and Γ are Lorentz oscillator amplitude and width, respectively. C_1 is a fitting parameter to ensure continuity of dielectric function at $E = e_t$. This modified Tauc-Lorentz model can be extended for the scenario of multiple oscillators, with the example discussed below.

Once the imaginary part of dielectric function can be available, the real portion ε_1 can be acquired based on Kramers-Kronig relation[1,12]:

$$\varepsilon_1 = 1 + \frac{1}{\pi}\beta\int_{-\infty}^{\infty}\frac{\varepsilon_2(\omega')}{\omega' - \omega}d\omega' = 1 + \frac{2}{\pi}\beta\int_{0}^{\infty}\frac{\omega'\varepsilon_2(\omega')}{\omega'^2 - \omega^2}d\omega' \tag{10}$$

where β denotes the principal value of the integral. By comparing experimental data acquired from small sized spherical nanoparticles with analytical solution of σ_{abs}, material- dependent dielectric function parameter ε_r can thus be determined.

In addition to the absorption, the CD response of a very small size chiral nanoparticles with spherical shape in a dielectric matrix (ε_m) can be expressed as[5,9]: $CD = CD_\xi + CD_\varepsilon$. The CD_ξ and CD_ε can be determined by:

$$\begin{cases} CD_\xi = A \bullet \mathrm{Im}(\xi) \bullet \dfrac{\mathrm{Re}(2\varepsilon_m + \varepsilon_r)}{|2\varepsilon_m + \varepsilon_r|^2} \\ CD_\varepsilon = -A \bullet \dfrac{\mathrm{Re}(\xi) \bullet \mathrm{Im}(\varepsilon_r)}{|2\varepsilon_m + \varepsilon_r|^2} \end{cases} \tag{11}$$

where $A = 8\omega\left|\bar{E}_0\right|^2 \varepsilon_m^{3/2} R^3$ is a constant and R is the radius of nanoparticle. Here, we can setup a multi-oscillator model for fitting:

$$\xi(\omega) = \sum_j \frac{a_j}{E - e_j + i\Gamma_j} \tag{12}$$

where a_j and Γ_j are the oscillator strength and the damping factor, respectively. Therefore, by comparing theoretical CD with experimental result of small sized chiral nanoparticle, chiral parameter, ξ can be therefore determined.

The parametric model as described above can be used to determine material-dependent parameters of optical function in a general manner, and the key is to utilize experimental data acquired from the spherical nanoparticles with very small size in order to improve fitting accuracy. We have used HgS as an example to illustrate our approach step-by-step to determine full set of parameters for computation of nanoparticles with achiral and chiral shape in current study, and we have also compared results from our approach with other techniques in order to substantiate our parametric modeling method in Supplementary Fig. 2. In order to validate our approach, we have synthesized small sized α-HgS nanoparticles with quasi-spherical morphology, and measured their absorption and CD spectra to compare with analytical expressions (see Supplementary Fig. 2b and 2c). According to Tauc-Lorentz model, the fitting imaginary part of dielectric function of HgS is modeled by two oscillators and expressed as following:

$$\varepsilon_2(E)=\begin{cases}\left(\frac{e_0}{E}\right)\bullet\frac{F\left(E-E_g\right)^2\Gamma}{\left(E^2-e_0^2\right)^2+\Gamma^2E^2}, & \left(E\geq e_{t1}\right)\\ \left(\frac{e_1}{E}\right)\bullet\exp\left(\frac{E-e_{t1}}{e_{u1}}\right), & \left(e_{t2}\leq E<e_{t1}\right)\\ \left(\frac{e_2}{E}\right)\bullet\left[0.93\exp\left(\frac{E-e_{t2}}{e_{u2}}\right)+0.07\right], & \left(e_{t3}\leq E<e_{t2}\right)\\ \left(\frac{e_3}{E}\right)\bullet\exp\left(\frac{E-e_{t3}}{e_{u3}}\right), & \left(e_{t4}\leq E<e_{t3}\right)\\ \left(\frac{e_4}{E}\right)\bullet\exp\left(\frac{E-e_{t4}}{e_{u4}}\right). & \left(0<E<e_{t4}\right)\end{cases}\qquad(13)$$

The optimized fitting parameters from the Tauc-Lorentz model are summarized in Supplementary Table 1. We also present our fitted dielectric function ε_1 and ε_2 in Supplementary Fig. 3d. We would like to point out that our fitted dielectric function agrees well with previous experimental values of cinnabar and parameters from DFT calculation[9,13]. Furthermore, we have presented fitted imaginary and real parts of chiral parameters of HgS in Supplementary Fig. 2e, which are also in the same range of values from previous literatures[9]. All these agreements together validate our parametric modeling method.

Comparison between simulation and experimental results

Given the full set of parameters acquired in Supplementary Eqs. 12 and 13, we have computed all nanostructures possessing different cooperative chirality, and compared with experimental data. We have mainly focused on addressing major CD features, such

as peak positions, relative intensity and overall variation tendency. To that, our simulations show good agreement with experimental results, which validate our computation model and parametric modeling method described above. Nevertheless, there exist some limitations of our current modeling and simulations as well as optical measurements, which should be improved in the future work: (i) In our simulations we have only considered single nanoparticle with size and twisting angle determined from statistical analysis of TEM images (i.e. averaged size and averaged twisting angle of nanoparticles acquired from statistical analysis are used to construct computation model. See for example Supplementary Table **3**). However, all optical measurements in our current work are ensemble measurements rather than single nanoparticle level characterization, in which many nanoparticles with inhomogeneous sample distribution have contributed to the CD and absorption signals. Such inhomogeneous sample variation in an ensemble measurement can contribute to the discrepancy between current computational and experimental results, and needs to be considered in the future simulation in order to fully reproduce some fine features manifested in ensemble measurements. From the experimental point of view, performing optical measurement at the single nanoparticle level can also avoid averaging effects caused by sample inhomogeneity; and (ii) The α-HgS is intrinsically an optically anisotropic material. However, in order to reduce computation cost for materials parametric fitting and simulation, our current work use isotropic dielectric function and chiral parameters, $\left(\xi,\ \varepsilon,\ \mu\right)$. Even though such simplification with isotropic fitting parameters has been extensive used in prior literature, future improvement of simulation by developing a full

set of anisotropic fitting parameters should be necessary in order to fully reproduce fine features of chiroptical response in experiment.

Supplementary Methods

All chemicals were used as received without further processing. D-penicillamine (D-pen, $C_5H_{11}NO_2S$, 99%), Mercury (II) nitrate monohydrate ($Hg(NO_3)_2 \bullet H_2O$, 98+%), and Thioacetamide (CH_3CSNH_2, 99%) were purchased from Acros. L-penicillamine (L-pen, $C_5H_{11}NO_2S$, 99%) and N-Acetyl-D-penicillamine ($C_7H_{13}NO_3S$, 99%) were purchased from Sigma-Aldrich. Sodium hydroxide (NaOH, pellets, 97%) was purchased from Fisher Scientific.

Deionized water (H_2O) was acquired by using Barnstead NANOPure water purification system, having a resistivity of 18.3 MΩ-cm. All aqueous solutions were prepared by using the deionized water.

Supplementary References

1. Jackson, J. D. *Classical Electrodynsmics*. (John Wiley & Sons, 1999)

2. Wang, B., Zhou, J., Koschny, T., Kafesaki, M. & Soukoulis, C. M. Chiral metamaterials: simulations and experiments. *J. Opt. A: Pure Appl. Opt.* **11**, 114003 (2009).

3. Ougier, S., Chenerie, I., Sihvola, A. & Priou, A. Propagation in bi-isotropic media: effect of different formalisms on the propagation analysis. *Prog. Electromagn. Res.* **9**, 19-30 (1994).

4. Lekner, J. Optical properties of isotropic chiral media. *Pure Appl. Opt.* **5**, 417-443 (1996).

5. Govorov, A. O. & Fan, Z. Theory of chiral plasmonic nanostructures comprising metal nanocrystals and chiral molecular media. *Chem.Phys.Chem* **13**, 2551-2560 (2012).

6. Ben-Moshe, A. *et al.* Enantioselective control of lattice and shape chirality in inorganic nanostructures using chiral biomolecules. *Nat. Commun.* **5**, 4302 (2014).

7. Novotny, L. & Hecht, B. *Principles of Nano-optics* (Cambridge University Press, 2012).

8. Bohren, C. F. & Huffman, D. R. *Absorption and Scattering of Light by Small Particles*. (Wiley-VCH, 2004).

9. Ben-Moshe, A., Govorov, A. O. & Markovich, G. Enantioselective synthesis of intrinsically chiral mercury sulfide nanocrystals. *Angew. Chem. Int. Ed.* **125**, 1275–1279 (2013).

10. Jellison Jr, G. E. & Modine, F. A. Parameterization of the optical functions of amorphous materials in the interband region. *Appl. Phys. Lett.* **69**, 371-373 (1996).

11. Ferlauto, A. S. *et al.* Analytical model for the optical functions of amorphous semi-conductors from the near-infrared to ultraviolet: Applications in thin film photo- voltaics. *J. Appl. Phys.* **92**, 2424-2436 (2002)

12. Fujiwara, H. *Spectroscopic Ellipsometry: Principles and Applications* (John Wiley & Sons, 2007)

13. Cardona, M. *et al.* Electronic and phononic properties of cinnabar: Ab initio calculations and some experimental results. *Phys. Rev. B* **82**, 085210 (2010).
